# Widespread inappropriate prescribing for older people with reduced kidney function: what are the harms and how do we tackle them? A scoping review for primary care

**DOI:** 10.1136/bmjqs-2025-018736

**Published:** 2025-10-07

**Authors:** Owen Thomas, Liz Glidewell, Sarah Alderson, David K Raynor, Su Wood

**Affiliations:** 1 Leeds Institute of Health Sciences (LIHS), University of Leeds, Leeds, UK; 2 Leeds Institute of Health Sciences, University of Leeds, Leeds, UK; 3 School of Healthcare, University of Leeds, Leeds, UK

**Keywords:** Primary care, Medication safety, General practice, Patient Safety, Pharmacoepidemiology

## Abstract

**Background:**

Increasing age is associated with reductions in kidney function and increasing polypharmacy. Most medicines are eliminated through the kidney, meaning older patients are at risk of medication accumulation and toxicity. This scoping review synthesised: (1) the prevalence at which older patients with reduced kidney function in primary care are exposed to inappropriate prescribing; (2) its associated harms; (3) the reasons for this occurring; and (4) the interventions used to improve prescribing practices.

**Methods:**

This scoping review searched ‘Medline’, ‘Embase’, ‘PsycINFO’, ‘CINAHL’ and ‘Web of Science’ for publications before October 2024. References were managed on EndNote V.X5 and thematic data analysis was undertaken on Microsoft Excel. Common themes were identified, summary statistics were calculated and insights were summarised through a narrative technique.

**Results:**

43 relevant studies explored the scale of inappropriate prescribing, estimating prevalences of patient exposure ranging from 0.6% to 49.1% (median 24.9%). Five studies explored the associated harm from inappropriate prescribing, but only one study assessed harm as a primary outcome. Eight studies that assessed difficulties in following prescribing guidelines in reduced kidney function suggested that a lack of awareness and trusted guidelines are fundamental problems. While 13 studies evaluated interventions for improving prescribing in reduced kidney function, only two demonstrated evidence of effectiveness and only one intervention was theoretically informed.

**Conclusions:**

Despite significant heterogeneity in study characteristics, it is clear that the prevalence of inappropriate prescribing for older people is uncomfortably high. There is a lack of evidence linking this to associated adverse outcomes, as well as identifying the causative issues driving this behaviour and the preventative interventions that could prevent harm.

**WHAT IS ALREADY KNOWN ON THIS TOPIC**

- Increasing age is associated with reduced kidney function and increased polypharmacy. Previous reviews have shown inappropriate prescribing in this population is prevalent in secondary care; however, the impact of this in primary care was not well understood.

**WHAT THIS STUDY ADDS**

- This review has clarified that inappropriate prescribing for older people with reduced kidney function is widespread throughout primary care settings, but the associated harms of this prescribing are poorly understood, and evidence for interventions that can prevent it is lacking.

**HOW THIS STUDY MIGHT AFFECT RESEARCH, PRACTICE OR POLICY**

- Given the ageing populations seen in many countries, awareness of this real and sizeable issue among those working in primary care will be crucial in efforts to reduce inappropriate prescribing. Those responsible for producing national and international guidelines should clarify how assessments of inappropriate prescribing in this population should be made (including the use of glomerular filtration rate estimating equations), and future research is needed to clarify who is at most risk of actual harm from such events, as well as what interventions can meaningfully reduce their occurrence.

##  Introduction and background

The majority of medicines are predictably eliminated through direct renal filtration at a rate proportional to the glomerular filtration rate (GFR).^1^ Patients with a reduced GFR are therefore at risk of medication accumulation, requiring dose adjustments or treatment cessation.^2^ Normal ageing produces variable declines in GFR from age 30 (1 mL/min/1.73 m^2^/year), which accelerates after age 65.^3^ Caution is advised when prescribing for older people due to complex age-related pharmacodynamic changes, such as altered body composition, receptor stimulation responses, albumin levels and homeostatic apparatus.^4 5^ Despite this, increasing age is associated with increasing polypharmacy. Across the European Union in 2019, 80.5% of those aged over 65 self-reported taking prescribed medication, compared with 21.5% of those aged 15–24.^6^

Measuring GFR directly is impractical, so multiple formulae exist to approximate it.^7^ The term ‘estimated GFR’ (eGFR) is reserved for the ‘Modification of Diet in Renal Disease’ (MDRD) or more recent ‘Chronic Kidney Disease Epidemiology Collaboration’ (CKD-EPI) equations, which were designed to assist chronic kidney disease (CKD) classification.^8^ The Cockcroft-Gault formula measures the creatinine clearance (CrCl) to approximate GFR and is used by pharmacokinetic studies for medication manufacturing and licensing decisions.^9^ These equations produce different results with increasing age; the Cockcroft-Gault formula may underestimate kidney function by 10%, while eGFR (MDRD/CKD-EPI) may overestimate kidney function by 29–69%.^10^ For 22% of older people, prescribing decisions would differ depending on the equation used and if weight is omitted or estimated during calculations.^11 12^ The British National Formulary (BNF) recently updated its guidance, advising that the Cockcroft-Gault formula should be used for dosing decisions in toxic medications and in older patients.^13^ Despite this, most English primary care practices still receive eGFR laboratory results without a Cockcroft-Gault CrCl.^12^

Older primary care patients have significantly higher odds than younger patients of being prescribed inappropriate medication doses for their kidney function.^12^ Harms relating to inappropriate prescribing in CKD are well documented in both primary and secondary care. A French study identified 467 hospital admissions for 360 patients due to inappropriate medication dosing in a nephrology outpatient setting (4.7 years, n=3033).^14^ A Japanese primary care study found that 40% of adults with CKD (mean age 57) received at least one inappropriate medication, while two or more inappropriate medications were associated with a 30% increased risk of eGFR decline.^15^ High prescribing rates in older people, combined with declining kidney function, GFR overestimation and concerns about resulting harm, pose a major challenge for primary care. This scoping review was designed to synthesise: the scale of the problem in primary care; the risk of harm to older patients; the reasons why it is difficult to follow prescribing guidelines in reduced kidney function; and the interventions evaluated to help reduce this inappropriate prescribing. Primary care has been defined here as any healthcare or prescribing activity taking place outside of a hospital or outpatient setting, including general practice, nursing homes and community pharmacies.

## Method

A scoping review framework was followed to ensure a robust and reproducible methodology, comprising: identifying research questions; finding and selecting appropriate studies; charting relevant data; and collating and summarising results.^16 17^ To identify research questions grounded in clinical practice, a case note review was undertaken of patients aged over 65 with a recorded reduced eGFR from five general practitioner (GP) practices (Bradford, UK: March–June 2010). For each case, the following were extracted: eGFR; calculated Cockcroft-Gault formula; repeat medications reviewed against BNF and Summary of Product Characteristics (SmPC) dosing guidance; and adverse drug reactions. Quantitative findings were interrogated using a mind map approach, which informed four research questions that lent themselves to a scoping review (online supplemental appendix I).^18^

Full strategies including specific search inclusion and exclusion criteria were developed for the four questions (online supplemental appendix II). For all questions, the following databases were searched for relevant studies: ‘Medline’, ‘Embase’, ‘PsycINFO’, ‘CINAHL’ and ‘Web of Science’. The initial searches were conducted in October 2015 by SW as part of a doctoral thesis; double screening was not undertaken at this stage.^18^ Two updates took place in January 2023 and October 2024 by OT and SW. EndNote V.X5 was used to manage citations. Search results pre-October 2015 were reviewed by SW based on title and abstract with duplicates removed; search updates were screened by OT with 10% double reviewed by SW; disagreements were settled by SA. Commentary pieces were excluded. Full-text copies pre-October 2015 were reviewed by SW; reasons for exclusion were not recorded. Full-text studies from update searches were reviewed by OT, with 10% of full-text studies double reviewed by SW; disagreements were settled by SA. Reasons for exclusion were recorded. Backward citation searches were undertaken manually for all relevant studies from all search phases to identify additional relevant studies; review articles underwent backward citation searching but were excluded themselves from the final analysis in favour of primary research studies.

Data were extracted into Microsoft Excel using a data extraction tool based on the review question and peer-reviewed criteria (online supplemental appendix III).^16^ Relevant ‘Critical Appraisals Skills Programme’ screening tools were used to appraise quality but not to exclude papers from review.^19^ Studies scoring <33% were considered low quality, those scoring <66% acceptable quality and higher scoring studies high quality.^20^ All data extraction was undertaken by OT, with 10% double reviewed by SW and disagreement settled by SA. The final studies included for each review question were evaluated for common themes; summary statistics were calculated; and insights were collated and summarised through a narrative technique.^21^ The research protocol was registered in September 2023 on ‘Inpalsy’, and this study has been reported to a validated checklist (online supplemental appendix IV).^22 23^

## Results

### Combined systematic search results

**Figure 1:**
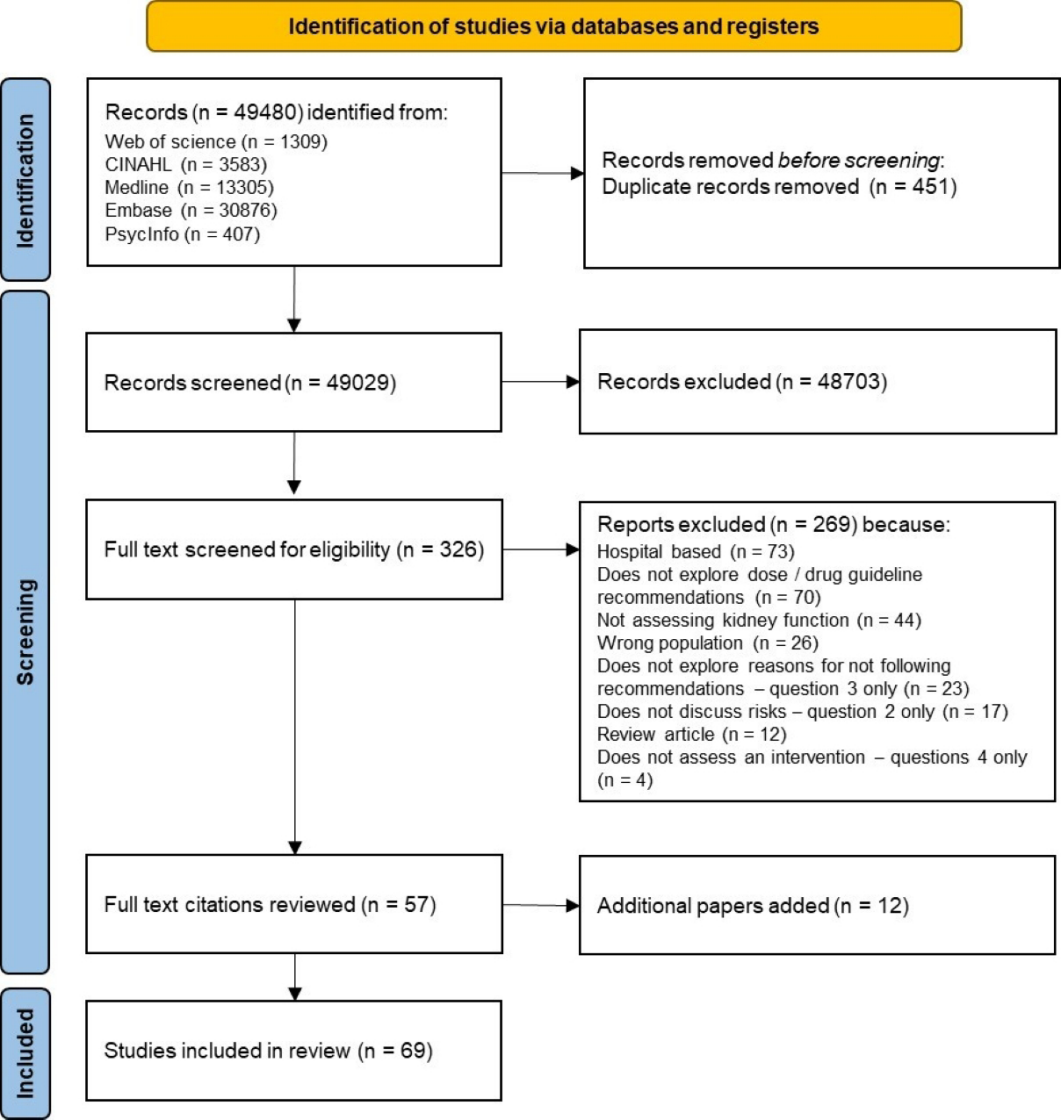
Preferred Reporting Items for Systematic Reviews and Meta-Analyses (PRISMA) diagram showing the combined systematic search process (adapted from Page *et al*^24^).

The combined search database returned 49 480 records. Duplicates were removed (n=451) and 48 703 records were rejected during abstract screening (figure 1). Full-text screening removed another 269 studies and backward citation review produced 12 additional studies, generating 69 studies for inclusion (online supplemental appendix V—review question-specific Preferred Reporting Items for Systematic Reviews and Meta-Analyses flow charts^24^). Some studies addressed more than one research question: three studies addressed questions 1 and 2^25^,^27^; eight studies addressed questions 1 and 4^28^,^35^; and one study addressed questions 3 and 4.^36^ Overall, 57 unique studies were identified (online supplemental appendix VI).

### What is the scale of the problem in primary care?

43 relevant studies were published between 2000 and 2023 (table 1; online supplemental appendix VI).^1225^,^35 37^ Publications originated from Europe (n=23, 54%),^1225^,^30 34 35 40 41 44 47 48 50^ North America (n=12, 28%),^3132 37^,^39 42 43 46 49 54 58 64^ Oceania (n=7, 16%)^33 45 53 56 59 61 65^ and Asia (n=1, 2.3%).^67^ Most study settings were in general primary care and the Veterans Association (n=34, 77%),^1225^,^30 32 34 38 40^ while others examined care homes (n=5, 12%),^31 33 37 39 47^ combined primary care and care home populations (n=1, 2%)^45^ and community pharmacy (n=1, 2%).^35^ Most studies included patients 65 years and older (n=22, 51%),^1226^,^28 30 32 34 37^ while this age group formed more than 85% total population in other studies. Most studies included all levels of kidney function (n=24, 56%),^1225^,^27 30 34 35 38^ while other studies selected those with pre-recorded reduced kidney function.^2931 33 35 42 47 50^,^54 56 57 59^ Most studies did not focus on specific comorbidities (n=30, 70%),^1225 27^,^29 31^ while others focused on patients with type 2 diabetes mellitus (T2DM) (n=5, 12%),^26 51 52 56 57^ atrial fibrillation (AF) (n=4, 9%),^49 60 62 65^ hypertension,^41^ polypharmacy,^30^ gout^50^ or a combination of these.^34^

**Table 1:** The prevalence of inappropriate prescribing for older people with reduced kidney function and the associated harms.

Question 1 - what is the scale of the problem in primary care?					
Principal author	Country and setting	Sample size	Participant characteristics	Overall prevalence	Medication or patient prevalence
Papaioannou *et al*^37^	Canada—care home	456	CKD; age >65	43.2%	Patient
Rothberg *et al*^38^	USA—primary care	814	Age >65	10.2%	Patient
Breton *et al*^25^	France—primary care	8701	Age >65	13.3%	Patient
Hanlon *et al*^39^	USA—VA care home	1304	Age >65	6.0%	Patient
Wood *et al*^40^	UK—primary care	594	Age >65	25.0%	Patient
Erler *et al*^28^	Germany—primary care	206	GFR <50; or age >70	34.5%	Patient
Schmidt-Mende *et al*^41^	Sweden—primary care	3345	CKD; age >65; hypertension	n/a	n/a
Durand *et al*^42^	USA—primary care	52	GFR <51	27.0%	Medication
Joosten *et al*^29^	Netherlands—primary care	1369	GFR <40	15.0%	Patient
Via-Sosa *et al*^30^	Spain—primary care	263	Age >65; polypharmacy	17.5%	Patient
Barnes *et al*^31^	USA—care home	146	CKD	7.2%	Medication
Farag *et al*^32^	Canada—primary care	1464	CKD 4–5; age >65	27.0%	Medication
Gheewala *et al*^33^	Australia—care home	323	CKD	8.7%	Patient
Steinman *et al*^43^	USA—VA primary care	462 405	Age >65	n/a	n/a
Van Pottelbergh *et al*^44^	Belgium—primary care	539	Age >80	8.2%	Patient
Khanal *et al*^45^	Australia—primary care and care home	4035	Age >65	28.1%	Patient
Chang *et al*^46^	USA—VA primary care	83 850	GFR 15–49; age >65	15.0%	Patient
Pourrat *et al*^34^	France—primary care	177	Age >65; hypertension or T2DM	24.9%	Patient
Becquemont *et al*^26^	France—primary care	588	Age >65; T2DM	21.9%	Patient
Hoffmann *et al*^47^	Germany—care home	685	Care home resident	19.7%	Both
Koster *et al*^48^	Netherlands—primary care	156	Age >65	0.6%	Patient
Parbtani and Dhindsa^49^	Canada—primary care	20	Age >75	40.0%	Patient
Pascart *et al*^50^	France—primary care	349	Gout	18.6%	Medication
Tebboth *et al*^51^	UK—primary care	3425	Age >40; gout	23.0%	Patient
Trifirò *et al*^52^	Italy—primary care	725	CKD; T2DM	32.5%	Patient
Khanal *et al*^53^	Australia—primary care	2628	n/a	12.9%	Medication
Guirguis-Blake *et al*^54^	USA—primary care	172	CKD 3–4	31.7%	Patient
Schmidt-Mende *et al*^55^	Sweden—primary care	32 533	CKD; age >65	42.5%	Patient
Wood *et al*^12^	UK—primary care	549 533	Age >65	n/a	n/a
Manski-Nankervis *et al*^56^	Australia—primary care	3505	CKD; T2DM	n/a	n/a
Spanopoulos *et al*^57^	UK—primary care	2580	T2DM	45.0%	Patient
Zhu *et al*^58^	Canada—primary care	3937	GFR <30; age >65	18.6%	Medication
Bezabhe *et al*^59^	Australia—primary care	44 259	CKD 3–4	n/a	n/a
Cardoso *et al*^60^	Portugal—primary care	772	AF	31.2%	Patient
Castelino *et al*^61^	Australia—primary care	48 731	CKD	35.0%	Patient
Ferrat *et al*^62^	France—primary care	1111	AF	n/a	n/a
MacRae *et al*^63^	UK—primary care	23 292	CKD	22.2%	Patient
Silva-Almodóvar *et al*^64^	USA—primary care	3624	CKD	33.0%	Patient
Troncoso-Mariño *et al*^27^	Spain—primary care	723 016	Age >65	11.1%	Patient
Bezabhe et al^65^	Australia—primary care	11 251	AF	n/a	n/a
Ramos *et al*^35^	Spain—community pharmacy	179	Age >60	39.1%	Patient
Ruiz-Boy *et al*^66^	Spain—primary care	273	CKD	49.1%	Patient
Naghnaghia *et al*^67^	Palestine—primary care	421	Age >60	36.8%	Patient

Question 2 - what is the risk of harm to older patients?					
Principal author	Country and setting	Sample size	Participant characteristics	Overall harm	Type of harm
Helldén *et al*^68^	Sweden—emergency	154	Age >65	OR 1493	Hospital admission
Breton *et al*^25^	France—primary care	8701	Age >65	Non-significant	Mortality (HR)
Becquemont *et al*^26^	France—primary care	588	Age >65; T2DM	Non-significant	Mortality (HR)
Alarkawi *et al*^69^	UK and Spain—primary care	320 578	GFR <45; age >40; osteoporosis	(-)30 to 0%	Mortality (HR)
Troncoso-Mariño *et al*^27^	Spain—primary care	723 016	Age >65	6–8%	Mortality (HR)

AF, atrial fibrillation; CKD, chronic kidney disease; GFR, glomerular filtration rate; T2DM, type 2 diabetes mellitus; VA, Veterans Affairs.

Full medication reviews were used to identify inappropriate medications and doses for an individual’s kidney function in most studies (n=25, 58%),^1225 27^,^31 33 35 38 40 41 43^ while others used lists of medications from trusted publications (n=5, 12%),^37 39 54 59 67^ direct oral anticoagulants (DOACs) alone (n=4, 9%),^49 60 62 65^ T2DM medications alone (n=4, 9%),^26 51 56 57^ antibiotics alone (n=3, 7%),^32 42 58^ a combination of hypertension and T2DM medications^34^ or colchicine alone.^50^ National guidelines were commonly used alone to identify inappropriate prescriptions based on kidney function (n=19, 44%),^1225 27 29^,^33 37 45 48 50 53 55 56 58 61 63 65^ while others used SmPC (n=9, 21%),^26 40 44 47 51 52 57 60 66^ expert opinions (n=8, 19%),^28 34 35 39 54 59 62 67^ Lexicomp (n=4, 9%),^42 43 46 64^ or did not state a guideline (n=3, 7%).^38 41 49^ The Cockcroft-Gault formula was used to estimate GFR in 14 studies (n=33%),^1231 37 40^,^42 45^ while other studies used MDRD (n=11, 26%),^2527 29 32^,^34 38 44 48 51 63^ CKD-EPI (n=8, 19%),^26 35 43 55 58 59 64 66^ a combination of these (n=6, 14%),^30 39 49 53 57 61^ or did not state a method (n=4, 9%).^28 54 56 67^ 28 studies excluded patients who did not have CKD or did not use the Cockcroft-Gault formula to estimate renal function (65%).^25^,^2729 31^ 18 studies were high quality (42%),^1225 26 34 37^,^39 46 50 52 53 55 59^ while 17 were acceptable quality (40%)^27^,^3032 40 41 43^ and eight were low quality (19%).^31 33 35 42 48 49 54 58^

**Figure 2:**
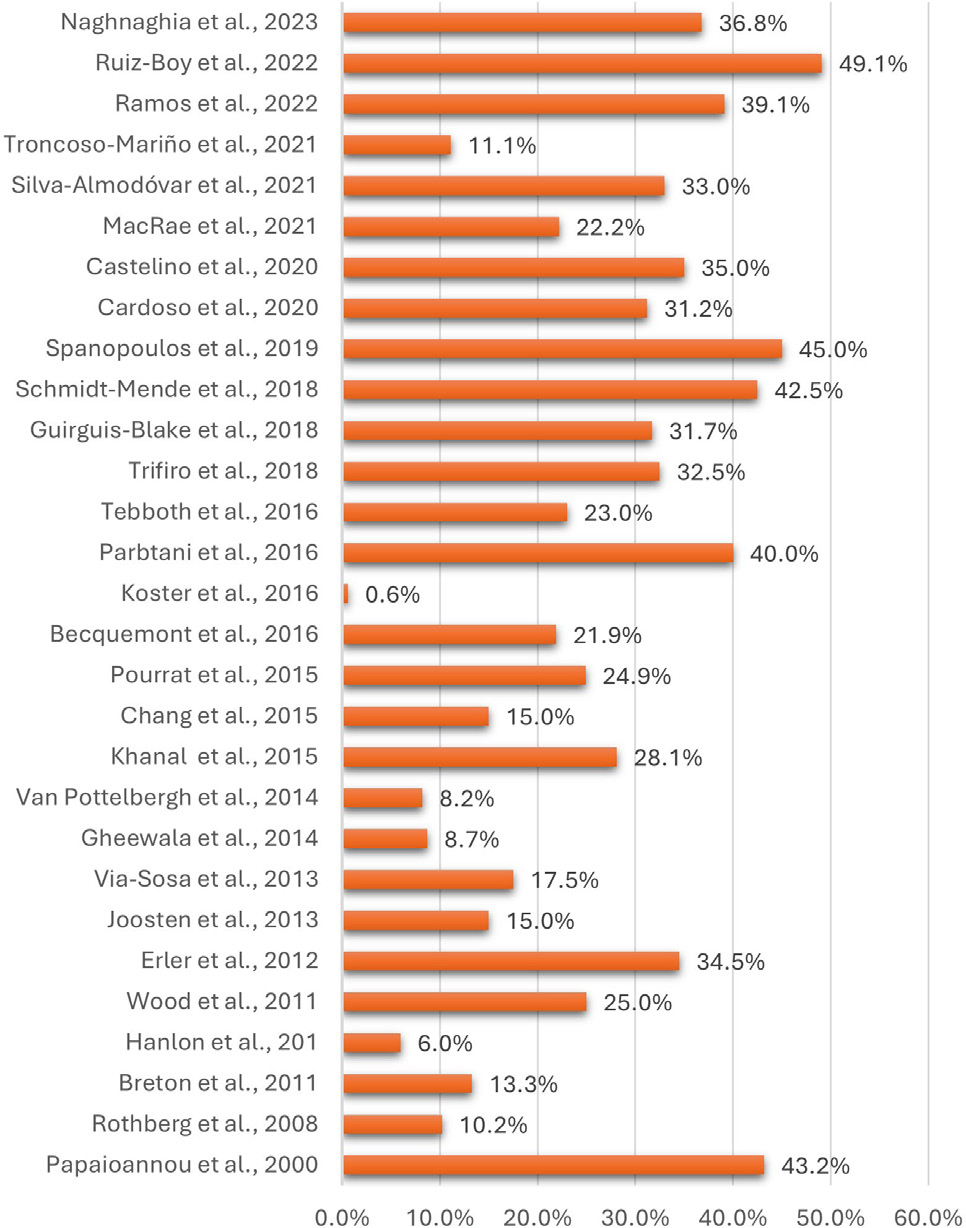
Prevalence of older primary care patients identified as being prescribed a drug dose that was inappropriate for their renal function.

**Figure 3:**
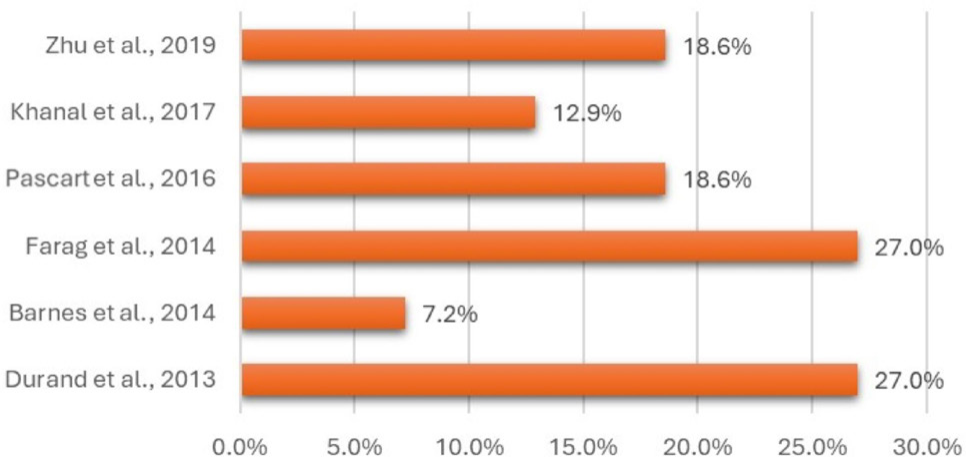
Prevalence of prescribed drugs for older primary care patients identified as being prescribed a drug dose that was inappropriate for the patient’s renal function.

Most studies calculated prevalence as ‘any individual having one or more medications currently prescribed inappropriately for their kidney function’ (n=30, 70%).^25^,^3033^ Sample sizes ranged from 20 to 723 016 patients (median 725; IQR 273–4035), while prevalence ranged from 0.6% to 49.1% (median 24.9%; IQR 13.3–35%) (figure 2). Other studies defined prevalence as ‘any medication prescription found to be prescribed at an inappropriate dose for an individual’s kidney function’ (n=7, 19%).^31 32 42 47 50 53 58^ Sample sizes ranged from 52 to 3937 medications (median 685; IQR 146–2628), while prevalence ranged from 3.9% to 27% (median 18.6%; IQR 7.2–27%) (figure 3). Seven studies did not look directly at overall prevalence and focused on providing insights into subgroups of populations and medications (table 1; online supplemental appendix VI).^12 41 43 56 59 62 65^

### What is the risk of harm to older patients?

Five relevant studies were identified, published exclusively in Europe between 2009 and 2021 (table 1; online supplemental appendix VI).^25^,^2768 69^ Study settings for four studies were in general primary care,^25^,^2769^ while one study looked at emergency department admissions.^68^ Four studies included adults aged over 65,^25^,^2768^ while one included adults aged over 40.^69^ Two studies constrained their study population to focus on patients with osteoporosis or T2DM,^26 69^ while one study selected patients with pre-recorded reduced kidney function (eGFR <45).^69^ Two studies used full medication reviews to identify inappropriate prescribing for an individual’s kidney function,^25 27^ while one study focused on metformin alone,^26^ one on bisphosphonates alone^69^ and one did not describe a strategy.^68^ National guidelines were used to identify inappropriate prescriptions based on kidney function in three studies,^25 27 68^ while SmPC was used in two studies.^26 69^ MDRD was used to estimate GFR in two studies,^25 27^ while one study used CKD-EPI,^26^ one used a combination of formulae^68^ and one did not state a method.^69^ Two studies were high quality,^26 27^ while three studies were acceptable quality.^25 68 69^

Harm caused by inappropriate prescribing was assessed through all-cause mortality using HRs in four studies.^25^,^2769^ Troncoso-Mariño *et al* used medication reviews in a retrospective cohort of older primary care patients and identified that exposure to inappropriate prescribing was associated with a higher risk of death (adjusted HR 1.08; 95% CI 1.06 to 1.09).^27^ Breton *et al* used medication reviews in a French primary care cohort of older patients and found a non-statistically significant association between mortality and inappropriate prescribing (HR 1.4; 95% CI 1.0 to 1.9).^25^ Becquemont *et al* looked at older French primary care patients with T2DM and found no increased death rate associated with inappropriate metformin dosing after adjusting for confounding.^26^ Alarkawi *et al* assessed mortality associated with bisphosphate use in Spanish and UK populations as their primary research question; they found improved mortality outcomes in the UK when using inappropriate bisphosphonate doses, but not in the Spanish sample.^69^ Helldén *et al* assessed the risk of hospital admission in a small sample of older patients presenting to the emergency department after inappropriate primary care prescribing, which identified a significant association (p=0.0001).^68^

### Why is it difficult to follow prescribing guidelines in reduced kidney function?

Eight studies were published between 1992 and 2022, originating from Europe (n=5, 63%),^1836 70^,^72^ North America (n=2, 25%)^73 74^ and Asia (n=1, 12%)^75^ (table 2; online supplemental appendix VI). Four studies used semistructured interviews (50%),^18 36 70 73^ three used surveys (38%)^71 72 75^ and one used mixed methods.^74^ Survey response sample sizes ranged from 50 to 233 (median 158; IQR 63.75–227.5). Interview samples ranged from 9 to 74 (median 16; IQR 10.5–59.75). Participants were often GPs (n=5, 63%),^1870^,^73^ while one study included pharmacists,^75^ one included GPs and nurses^36^ and one included all primary care clinical staff, endocrinologists and general internists.^74^ Most studies focused on prescribing in reduced kidney function (n=5, 63%),^18 36 71 74 75^ while others explored general prescribing (n=3, 37%).^70 72 73^ One study was high quality,^18^ while others were acceptable (n=5, 63%)^7071 73^,^75^ or low quality (n=2, 25%).^36 72^

**Table 2:** What challenges exist in following prescribing guidelines for patients with reduced kidney function and what strategies have been shown to improve prescribing?

Question 3 - why is it difficult to follow prescribing guidelines in reduced kidney function?			
Principal author	Country and participant type	Study type	Themes
Bradley^70^	UK—GP	Semistructured interviews	Balancing other considerations; lack of awareness; concern about toxicity
Jonville-Béra *et al*^71^	France—GP	Qualitative survey	Concerns about accessing/trusting/using guidelines in primary care; lack of awareness
Wood^18^	UK—GP	Semistructured interviews	Lack of awareness; confusion regarding different kidney function formulae; balancing other considerations; warning overload; concerns about accessing/trusting/using guidelines in primary care; concern about toxicity
Schmidt-Mende^36^	Sweden—GP; nurse	Semistructured interviews	Complexity in prescribing in the elderly; concerns about accessing/trusting/using guidelines in primary care; threats to GP autonomy
Teh and Lee^75^	Malaysia—pharmacist	Qualitative survey	Concerns about accessing/trusting/using guidelines in primary care; lack of awareness
Campbell *et al*^73^	Canada—GP	Semistructured interviews	Confusion regarding different kidney function formulae; confusion about prescribing in the elderly; lack of awareness
Allouchery *et al*^72^	France—GP	Qualitative survey	Lack of awareness; improvement due to external support
Flory *et al*^74^	USA—mixed primary and secondary care	Semistructured interviews and qualitative survey	Concerns about accessing/trusting/using guidelines in primary care; concerns about toxicity; lack of awareness

Question 4 - what has been shown to help improve prescribing in reduced kidney function?				
Principal author	Country and setting	Sample size	Patient characteristics	Intervention effect
Field *et al*^79^	Canada—care home	833	Taking a medication from a list of risky medications	Positive trend
Erler *et al*^28^	Germany—primary care	404	GFR <50 or age >70; any medication	Positive
Geerts *et al*^76^	Netherlands—primary care	650	Age >70; diabetes or CVD; taking a medication from a list of diabetic and CVD medications	Positive trend
Joosten *et al*^29^	Netherlands—primary care	1369	Any medication	Positive trend
Via-Sosa *et al*^30^	Spain—community pharmacy	354	Age >65; polypharmacy; any medication	Positive
Barnes *et al*^31^	USA—care home	146	CKD; any medication	Positive trend
Farag *et al*^32^	Canada—primary care	1464	CKD 4/5; age >65; taking a medication from a list of antibiotics	Neutral
Gheewala *et al*^33^	Australia—care home	323	Any medication	Positive trend
Pourrat *et al*^34^	Community pharmacy	180	Age >65; diabetes or hypertension; any medication	Positive trend
Keohane *et al*^77^	France—primary care	158	Taking an NSAID	Positive trend
Schmidt-Mende^36^	Sweden—primary care	69	Age >65; taking a medication from the “Screening Tool of Older Persons' Prescriptions / Screening Tool to Alert to Right Treatment” (STOPP/START) criteria V2	Neutral
Cámara Ramos *et al*^35^	Spain—community pharmacy	179	Age >60; any medication	Positive trend
Jones and Patel^78^	UK—primary care	577	AF; taking a DOAC	Positive trend

AF, atrial fibrillation; CKD, chronic kidney disease; CVD, cardiovascular disease; DOAC, direct oral anticoagulant; GFR, glomerular filtration rate; GP, general practice; NSAID, non-steroidal anti-inflammatory drug.

The common themes described included a lack of awareness about inappropriate prescribing in primary care (n=7, 88%)^1870^,^75^ and concerns from clinicians about not being able to access, trust or apply guidelines in a pragmatic way within primary care (n=7, 88%).^1836 70 71 73^,^75^ Less common themes included concerns around: toxicity in kidney dysfunction prescribing (n=3, 38%)^18 70 74^; balancing other considerations in time-pressured settings (n=2, 25%)^18 70^; different kidney function formula (n=2, 25%)^18 73^; complexity when prescribing in the elderly with kidney dysfunction (n=2, 25%)^36 73^; warning overload from computer alert systems (n=1, 13%)^18^; guidelines threatening GP autonomy (n=1, 13%)^36^; and external support being needed to produce improvement (n=1, 13%)^72^ (table 2).

### What has been shown to help improve prescribing in reduced kidney function?

13 relevant studies were published between 2009 and 2023, originating from Europe (n=9, 69%),^2930 32 34^,^36 76^ North America (n=3, 23%)^31 32 79^ and Oceania (n=1, 8%)^33^ (table 2; online supplemental appendix VI). Most study settings were in primary care (n=7, 54%),^2829 32 36 76^,^78^ while others were in care homes (n=3, 23%)^31 33 79^ and community pharmacies (n=3, 23%).^30 34 35^ Three studies focused on patients with prespecified reduced kidney function (23%).^28 31 32^ Most studies looked at populations aged 65 and older (n=7, 54%),^2830 32 34^,^36 76^ while others had more than 85% of their population aged 65 or older (n=6, 46%).^2931 33 77^,^79^ Most studies did not select for patients diagnosed with comorbidities (n=9, 69%),^2829 31^,^33 35 36 77 79^ while one study included patients with T2DM or cardiovascular disease,^76^ one selected for polypharmacy,^30^ one included patients with T2DM or hypertension^34^ and one included patients with AF.^78^ Most studies assessed all prescribed medications (n=7, 54%),^28^,^3133^ while four studies used predefined medication lists (31%),^32 36 76 79^ one assessed non-steroidal anti-inflammatory drugs only (8%)^77^ and one assessed DOACs only (8%).^78^ National guidelines were used to assess if prescribing was inappropriate for the kidney function in seven studies (54%),^29^,^3376 79^ while four studies used expert opinion (31%)^2834^,^36^ and two did not state how decisions were made (15%).^77 78^ MDRD was used to estimate GFR in five studies (39%),^29 32 34 76 77^ while four studies used the Cockcroft-Gault formula (31%),^31 33 78 79^ two used CKD-EPI (15%),^35 36^ one used a combination of formulae (8%)^30^ and one did not state a method (8%).^28^

The most common primary intervention employed pharmacist medication reviews (n=5, 39%),^3031 33^,^35^ while four studies used Computer Decision Support Systems (CDSS) (31%),^28 29 77 79^ two used a combination of pharmacist medication review and CDSS (15%),^76 78^ one introduced standard laboratory eGFR reporting (8%)^32^ and one used clinician education (8%).^36^ While most studies did not use additional secondary interventions (n=5; 39%),^31^,^3376 79^ three studies used multifaceted interventions (24%),^28 36 78^ two used additional clinician education (15%),^30 34^ one used a medication alert system (8%),^77^ one used audit and feedback (8%)^29^ and one used point-of-care testing (8%).^35^

Intervention effectiveness was often evaluated through single-arm intervention studies (n=5, 39%),^2933^,^35 76^ while two studies used before-and-after observational designs (15%),^31 77^ two used time-series analysis (15%)^32 78^ and one used historical controls (8%).^30^ Only three studies used randomised controlled trial (RCT) designs (23%).^28 36 79^ Study quality was mostly low (n=6, 46%)^3133 35 76^,^78^ or adequate (n=6, 46%),^28 29 32 34 36 79^ with one high-quality study identified (8%).^30^ Due to the inherent traits of observational, non-randomised and pseudorandomised designs, eight studies were unable to produce strong evidence on effectiveness (62%).^2931 33^,^35 76^ Five studies were suitable to provide evidence (31%), of which two showed no intervention effect, one showed a non-statistically significant favourable association and two showed evidence of intervention effectiveness. Erler *et al* used an RCT to evaluate a CDSS alongside a multifaceted intervention involving education, checklists and leaflets to reduce inappropriate prescribing in German primary care (eGFR <50 or aged >70); the intervention group had 0.46 times the odds of having more than one prescription exceeding recommended maximum dose compared with controls (95% CI 0.26 to 0.82; p=0.008).^28^ Via-Sosa *et al* compared pharmacist medication reviews to historical controls in older patients taking three or more medications attending Spanish community pharmacies; the difference in inappropriately dosed medication between control and intervention groups was 0.73% before intervention and 13.5% after intervention (p<0.001).^30^ Field *et al* used a CDSS to reduce inappropriate prescribing for Canadian care home patients and found a non-statistically significant increase in CDSS-related medication order appropriateness.^79^ Farag *et al* used a time-series analysis to evaluate mandatory eGFR laboratory reporting for older primary care patients with CKD 4–5 when prescribed antibiotics; no improvement in inappropriate prescribing was identified.^32^ Schmidt-Mende used an RCT to test a multifaceted educational intervention for older patients in Swedish primary care to reduce inappropriate prescribing but found no difference between intervention groups (22.0% vs 22.8%).^36^ Only one study (8%) referenced a theoretical basis for their intervention (the clinical audit model), which they evaluated through a before-and-after study design.^77^

## Discussion

Increasing age is associated with reductions in kidney function and increasing polypharmacy.^80 81^ With half of all medication being prescribed by primary care, the potential for age and kidney function to be significant risk factors in primary care for inappropriate prescribing is substantial.^40 82^ This review highlights the prevalence of this inappropriate prescribing, alongside its associated harms, causes and interventions that have been evaluated to reduce its impact.

The validity of this scoping review is strengthened by the involvement of experienced researchers actively engaged in pharmacy, primary care and academia, which has addressed research questions grounded within clinical practice. The methodology adhered to the study protocol published prior to update searches and followed a refined framework first proposed by Arksey and O’Malley.^16 17 23^ Multiple databases were searched, including backward citation searching of relevant studies. Broad research questions were deliberately designed to be applicable across different healthcare contexts and time periods, allowing questions to remain relevant despite an evolving primary care landscape with changing clinical prescribing roles and health technology.

Despite these strengths, this review has limitations. The initial searches were conducted as part of SW’s doctoral thesis without double screening.^18^ Partial double screening (10%) was implemented for update searches to mitigate selection bias and enhance reliability. Despite these adjustments, the absence of full double screening may have increased the risk of missing relevant studies. Results should be interpreted in the context of language limitations placed on the search criteria and with the understanding that clinical primary care may differ from research settings. Due to the broad scoping review methodology and the inclusion of populations not exclusively composed of older adults, studies were too heterogeneous for meta-analysis evaluation. However, the inclusion of this broad range of studies enabled a richer and more comprehensive overview of the topics, producing valuable insights into the prevalence, impacts and underlying causes of this inappropriate prescribing.

The lack of primary care research on compliance with recommended dosing guidelines in reduced kidney function was first highlighted in 2004.^83^ This review demonstrates a significant expansion in research addressing this concern. In 2017, a systematic review focusing on secondary care raised concerns over the plethora of different methodologies used to investigate these concerns.^84^ This review demonstrates that similar significant heterogeneity exists within primary care research, with very few studies investigating the same population in terms of age, kidney function or comorbidity. Of particular concern is the lack of agreement concerning GFR estimating formulae used to make dosing decisions, which likely reflects ongoing confusion throughout clinical practice.^12^ A 2019 UK regulatory alert highlighted reports documenting patient harm due to incorrect primary care dosing for elderly patients with reduced kidney function, yet this review shows that the scale and economic impact of these harms remain poorly understood.^85^ Nearly two-thirds of studies identified here excluded those without CKD or did not use the Cockcroft-Gault formula, potentially overlooking older patients with an acceptable eGFR but a lower and more accurate Cockcroft-Gault CrCl. A multitude of guidelines and medication review techniques were used, making comparison challenging and potentially explaining some of the wide variation in practice identified. This corresponds with themes summarised here, suggesting that prescribers struggle to confidently find relevant and consistent primary care guidelines. Despite this heterogeneity, a number of high-quality studies were identified. Most focused on the prevalence of older people with one or more medications prescribed inappropriately in relation to kidney function (median prevalence 24.9%). In comparison, evidence of associated harms in primary care from this was notably limited and of variable quality. All-cause mortality was most commonly analysed, but only one study made this their primary research question.^69^

A range of interventions were used to support primary care clinicians to reduce inappropriate prescribing, which usually incorporated pharmacist medication reviews or CDSS. Most interventions were not evaluated using robust methodology and only one study described a theoretical model underpinning the intervention’s mechanism of action, despite these being core implementation research recommendations.^86^ These conclusions are similar to those from a 2017 review that focused on inpatient and outpatient populations of all ages with reduced kidney function.^87^ Several factors identified by this study may help explain why inadequate progress has been made on this important clinical issue. The complexity of prescribing for older people with reduced kidney function appears to often exceed the scope of standard clinical training, leading to a lack of confidence among non-specialist prescribers in primary care. The use of pharmacist medication reviews seen here may reflect these concerns, with pharmacists being perceived to have more relevant specialist expertise. This is consistent with findings from a UK-based study exploring primary care medication reviews, where clinicians felt that pharmacist reviews were more thorough than those by GPs.^88^ Their widespread use may be limited by perceived concerns of their resource-intensive nature, a concern that is prevalent given global challenges to healthcare budgets.^88^ A broader scoping review of pharmacy-led medication reviews showed that most related research was observational, with minimal exploration of clinical effectiveness or cost-effectiveness.^89^ CDSS offers a scalable solution but may struggle to overcome issues in implementation due to the need to adapt to multiple healthcare software packages, incorporate a wide variety of patient contexts and alert fatigue avoidance.^90 91^

Prescribing safely for older people with complex age-related diseases that impact pharmacodynamics requires a nuanced, evidence-based approach, yet the wider problem persists of a lack of fundamental under-representation of this population in trials that can clarify and quantify the risks posed to the highest users of these medications.^92^ The issues around the application of different formulae for approximating GFR exemplify this issue. Valid concerns over increased risk to older people from clinical trial participation and impacts on trial designs need to be overcome given the changing global population age pyramid.^92^

## Conclusions

Inappropriate prescribing for older people with reduced kidney function in primary care is highly prevalent. Despite this, there is limited research on its associated harms. Primary care clinicians may be ill equipped to correct such inappropriate prescribing, and interventions proposed to support clinicians have not been rigorously evaluated at scale.

This study recommends that those working in clinical practice must recognise the real and sizeable prevalence of inappropriate prescribing within primary care for older people with reduced kidney function. In order to facilitate this, those responsible for producing national and international guidelines should clarify how assessments of kidney function and inappropriate prescribing in this population should be made. Future research is needed to clarify who is at most risk of actual harm from such events. While observational research has a role to play in intervention development, there is a clear and present need for research that provides robust evidence for the effectiveness of interventions, which should be theoretically informed to change behaviours and reduce the prevalence of inappropriate prescribing for this population.

## Supplementary material

10.1136/bmjqs-2025-018736 online supplemental appendix 1

## Data Availability

All data relevant to the study are included in the article or uploaded as supplementary information.

## References

[1] Brenner G, Stevens C (2017). Brenner and Stevens’ pharmacology.

[2] Brater DC (2002). Measurement of renal function during drug development. Brit J Clinical Pharma.

[3] Glassock RJ, Winearls C (2009). Ageing and the glomerular filtration rate: truths and consequences. Trans Am Clin Climatol Assoc.

[4] Ponticelli C, Sala G, Glassock RJ (2015). Drug management in the elderly adult with chronic kidney disease: a review for the primary care physician. Mayo Clin Proc.

[5] Ashley C, Dunleavy A (2018). The renal drug handbook: the ultimate prescribing guide for renal practitioners.

[6] Eurostat (2022). Self-reported use of prescribed medicines by sex, age and educational attainment level.

[7] Boron WF, Boulpaep EL (2016). Medical physiology e-book.

[8] Levey AS, Stevens LA, Schmid CH (2009). A new equation to estimate glomerular filtration rate. Ann Intern Med.

[9] Helou R (2010). Should we continue to use the Cockcroft-Gault formula?. Nephron Clin Pract.

[10] Roberts GW, Ibsen PM, Schiøler CT (2009). Modified diet in renal disease method overestimates renal function in selected elderly patients. Age Ageing.

[11] von Scholten BJ, Persson F, Svane MS (2017). Effect of large weight reductions on measured and estimated kidney function. BMC Nephrol.

[12] Wood S, Petty D, Glidewell L (2018). Application of prescribing recommendations in older people with reduced kidney function: a cross-sectional study in general practice. Br J Gen Pract.

[13] Joint Formulary Committee Prescribing in renal impairment.

[14] Laville SM, Gras-Champel V, Hamroun A (2024). Kidney Function Decline and Serious Adverse Drug Reactions in Patients With CKD. Am J Kidney Dis.

[15] Kimura H, Yoshida S, Takeuchi M (2023). Impact of Potentially Inappropriate Medications on Kidney Function in Chronic Kidney Disease: Retrospective Cohort Study. Nephron.

[16] Arksey H, O’Malley L (2005). Scoping studies: towards a methodological framework. Int J Soc Res Methodol.

[17] Levac D, Colquhoun H, O’Brien KK (2010). Scoping studies: advancing the methodology. Implement Sci.

[18] Wood SI (2016). Are recommendations for prescribing applied for older people with reduced kidney function in primary care? a mixed methods study to explore and improve implementation.

[19] CASP UK - OAP Ltd CASP checklists - critical appraisal skills programme. casp - crit. apprais. ski. programme. https://casp-uk.net/casp-tools-checklists/.

[20] CASP FAQ’s. casp - crit. apprais. ski. programme. https://casp-uk.net/faqs/.

[21] Gilbert E, Turner M, de Viggiani N (2022). Developing a typology of models of palliative care delivery in prisons in high-income countries: protocol for a scoping review with narrative synthesis. BMJ Open.

[22] Tricco AC, Lillie E, Zarin W (2018). PRISMA Extension for Scoping Reviews (PRISMA-ScR): Checklist and Explanation. Ann Intern Med.

[23] Thomas O, Alderson S, Wood S (2023). Inplasy Protocol 5292 - Prescribing for older people with reduced kidney function in primary care – a systematic scoping literature review. INPLASY.

[24] Page MJ, McKenzie JE, Bossuyt PM (2021). The PRISMA 2020 statement: an updated guideline for reporting systematic reviews. BMJ.

[25] Breton G, Froissart M, Janus N (2011). Inappropriate drug use and mortality in community-dwelling elderly with impaired kidney function--the Three-City population-based study. Nephrol Dial Transplant.

[26] Becquemont L, Bauduceau B, Benattar-Zibi L (2016). Cardiovascular Drugs and Metformin Drug Dosage According to Renal Function in Non-Institutionalized Elderly Patients. Basic Clin Pharmacol Toxicol.

[27] Troncoso-Mariño A, López-Jiménez T, Roso-Llorach A (2021). Medication-related problems in older people in Catalonia: A real-world data study. Pharmacoepidemiol Drug Saf.

[28] Erler A, Beyer M, Petersen JJ (2012). How to improve drug dosing for patients with renal impairment in primary care - a cluster-randomized controlled trial. BMC Fam Pract.

[29] Joosten H, Drion I, Boogerd KJ (2013). Optimising drug prescribing and dispensing in subjects at risk for drug errors due to renal impairment: improving drug safety in primary healthcare by low eGFR alerts. BMJ Open.

[30] Via-Sosa MA, Lopes N, March M (2013). Effectiveness of a drug dosing service provided by community pharmacists in polymedicated elderly patients with renal impairment--a comparative study. BMC Fam Pract.

[31] Barnes KD, Tayal NH, Lehman AM (2014). Pharmacist-driven renal medication dosing intervention in a primary care patient-centered medical home. Pharmacotherapy.

[32] Farag A, Garg AX, Li L (2014). Dosing Errors in Prescribed Antibiotics for Older Persons With CKD: A Retrospective Time Series Analysis. Am J Kidney Dis.

[33] Gheewala PA, Peterson GM, Curtain CM (2014). Impact of the pharmacist medication review services on drug-related problems and potentially inappropriate prescribing of renally cleared medications in residents of aged care facilities. Drugs Aging.

[34] Pourrat X, Sipert A-S, Gatault P (2015). Community pharmacist intervention in patients with renal impairment. Int J Clin Pharm.

[35] Cámara Ramos I, Escribá Martí G, Escudero Quesada V (2022). MO378: The Importance of Community Pharmacy in Chronic Kidney Disease Patient Management. Drug Dosage Adjustment and Nephrotoxicity Detection. Nephrol Dial Transplant.

[36] Schmidt-Mende K (2019). Every coin has two sides: the challenge of addressing inappropriate prescribing in older patients in primary care.

[37] Papaioannou A, Clarke JA, Campbell G (2000). Assessment of adherence to renal dosing guidelines in long-term care facilities. J Am Geriatr Soc.

[38] Rothberg MB, Kehoe ED, Courtemanche AL (2008). Recognition and Management of Chronic Kidney Disease in an Elderly Ambulatory Population. J GEN INTERN MED.

[39] Hanlon JT, Wang X, Handler SM (2011). Potentially Inappropriate Prescribing of Primarily Renally Cleared Medications for Older Veterans Affairs Nursing Home Patients. J Am Med Dir Assoc.

[40] Wood S, Petty D, Glidewell L (2011). Are we over-dosing our elderly patients with renally excreted drugs in primary care?. Int J Pharm Pract.

[41] Schmidt-Mende K, Wettermark B, Andersen M (2012). Potentially inappropriate drugs in elderly hypertensive patients with impaired renal function. Pharmacoepidemiol DRUG Saf.

[42] Durand CR, Carr RJ, Boudreau SM (2013). Compliance with Renal Dosing Guidelines for Outpatient Antiinfective Prescriptions. Journal of Pharmacy Technology.

[43] Steinman MA, Miao Y, Boscardin WJ (2014). Prescribing quality in older veterans: a multifocal approach. J Gen Intern Med.

[44] Van Pottelbergh G, Mertens A, Azermai M (2014). Drug prescriptions unadapted to the renal function in patients aged 80 years and older. Eur J Gen Pract.

[45] Khanal A, Peterson GM, Castelino RL (2015). Potentially inappropriate prescribing of renally cleared drugs in elderly patients in community and aged care settings. Drugs Aging.

[46] Chang F, O’Hare AM, Miao Y (2015). Use of Renally Inappropriate Medications in Older Veterans: A National Study. J Am Geriatr Soc.

[47] Hoffmann F, Boeschen D, Dörks M (2016). Renal Insufficiency and Medication in Nursing Home Residents. A Cross-Sectional Study (IMREN). Dtsch Arztebl Int.

[48] Koster ES, Philbert D, Noordam M (2016). Availability of information on renal function in Dutch community pharmacies. Int J Clin Pharm.

[49] Parbtani A, Dhindsa G (2016). An eGFR obtained by MDRD equation can potentially compromise anticoagulant dosing safety in elderly patients with atrial fibrillation. Can Fam Physician.

[50] Pascart T, Lancrenon S, Lanz S (2016). GOSPEL 2 - Colchicine for the treatment of gout flares in France - a GOSPEL survey subgroup analysis. Doses used in common practices regardless of renal impairment and age. Joint Bone Spine.

[51] Tebboth A, Lee S, Scowcroft A (2016). Demographic and Clinical Characteristics of Patients With Type 2 Diabetes Mellitus Initiating Dipeptidyl Peptidase 4 Inhibitors: A Retrospective Study of UK General Practice. Clin Ther.

[52] Trifirò G, Parrino F, Pizzimenti V (2016). The Management of Diabetes Mellitus in Patients with Chronic Kidney Disease: A Population-Based Study in Southern Italy. Clin Drug Investig.

[53] Khanal A, Peterson GM, Jose MD (2017). Comparison of equations for dosing of medications in renal impairment. Nephrology (Carlton).

[54] Guirguis-Blake J, Keppel GA, Holmes J (2018). Prescription of high-risk medications among patients with chronic kidney disease: a cross-sectional study from the Washington, Wyoming, Alaska, Montana and Idaho region Practice and Research Network. Fam Pract.

[55] Schmidt-Mende K, Wettermark B, Andersen M (2019). Prevalence of renally inappropriate medicines in older people with renal impairment - A cross-sectional register-based study in a large primary care population. Basic Clin Pharmacol Toxicol.

[56] Manski-Nankervis J-AE, Thuraisingam S, Sluggett JK (2019). Prescribing for people with type 2 diabetes and renal impairment in Australian general practice: A national cross sectional study. Prim Care Diabetes.

[57] Spanopoulos D, Busse M, Webb J (2019). DPP-4 Inhibitor Dose Selection According to Manufacturer Specifications: A Contemporary Experience From UK General Practice. Clin Ther.

[58] Zhu JXG, Nash DM, McArthur E (2019). Nephrology comanagement and the quality of antibiotic prescribing in primary care for patients with chronic kidney disease: a retrospective cross-sectional study. Nephrol Dial Transplant.

[59] Bezabhe WM, Kitsos A, Saunder T (2020). Medication Prescribing Quality in Australian Primary Care Patients with Chronic Kidney Disease. J Clin Med.

[60] Cardoso CS, Sousa JA, Simões P (2020). Misdosing of Non-Vitamin K Antagonist Oral Anticoagulants in Primary Care. Clin Ther.

[61] Castelino RL, Saunder T, Kitsos A (2020). Quality use of medicines in patients with chronic kidney disease. BMC Nephrol.

[62] Ferrat E, Fabre J, Galletout P (2021). Inappropriate direct oral anticoagulant prescriptions in patients with non-valvular atrial fibrillation: cross-sectional analysis of the French CACAO cohort study in primary care. *Br J Gen Pract*.

[63] MacRae C, Mercer S, Guthrie B (2021). Potentially inappropriate primary care prescribing in people with chronic kidney disease: a cross-sectional analysis of a large population cohort. Br J Gen Pract.

[64] Silva-Almodóvar A, Hackim E, Wolk H (2021). Potentially Inappropriately Prescribed Medications Among Medicare Medication Therapy Management Eligible Patients with Chronic Kidney Disease: an Observational Analysis. J Gen Intern Med.

[65] Bezabhe WM, Bereznicki LR, Radford J (2022). Comparing the renal outcomes in patients with atrial fibrillation receiving different oral anticoagulants. Expert Rev Clin Pharmacol.

[66] Ruiz-Boy S, Rodriguez-Reyes M, Clos-Soldevila J (2022). Appropriateness of drug prescriptions in patients with chronic kidney disease in primary care: a double-center retrospective study. BMC Prim Care.

[67] Naghnaghia S, Nazzal Z, Abu Alya L (2023). The association between renal impairment and polypharmacy among older Palestinian patients: a multi-center cross-sectional study. BMC Prim Care.

[68] Helldén A, Bergman U, von Euler M (2009). Adverse drug reactions and impaired renal function in elderly patients admitted to the emergency department: a retrospective study. Drugs Aging.

[69] Alarkawi D, Ali MS, Bliuc D (2020). Oral Bisphosphonate Use and All-Cause Mortality in Patients With Moderate-Severe (Grade 3B-5D) Chronic Kidney Disease: A Population-Based Cohort Study. J Bone Miner Res.

[70] Bradley CP (1992). Uncomfortable prescribing decisions: a critical incident study. BMJ.

[71] Jonville-Béra AP, Paroux L, Autret-Leca E (2008). Assessing general practitioners’ prescribing behaviour in elderly patients with concealed renal failure. Br J Clin Pharmacol.

[72] Allouchery C, Bene J, Bodein I (2021). Metformin-related lactic acidosis: Knowledge of risk factors and prevention by General Practitioners in Nord and Pas-de-Calais departments (France). Fundam Clin Pharmacol.

[73] Campbell DJT, Lee-Krueger RCW, McBrien K (2020). Strategies for enhancing the initiation of cholesterol lowering medication among patients at high cardiovascular disease risk: a qualitative descriptive exploration of patient and general practitioners’ perspectives on a facilitated relay intervention in Alberta, Canada. BMJ Open.

[74] Flory JH, Guelce D, Goytia C (2023). Prescriber Uncertainty as Opportunity to Improve Care of Type 2 Diabetes with Chronic Kidney Disease: Mixed Methods Study. J Gen Intern Med.

[75] Teh XR, Lee SWH (2019). Pharmacists’ attitude, self‐reported knowledge and practice of dosage adjustment among chronic kidney disease patients in Malaysia. Pharmacy Practice and Res.

[76] Geerts AFJ, Scherpbier-de Haan ND, de Koning FHP (2012). A pharmacy medication alert system based on renal function in older patients. Br J Gen Pract.

[77] Keohane DM, Dennehy T, Keohane KP (2017). Reducing inappropriate non-steroidal anti-inflammatory prescription in primary care patients with chronic kidney disease. Int J Health Care Qual Assur.

[78] Jones T, Patel V (2023). 93 Improving the monitoring of renal function in patients with atrial fibrillation prescribed direct oral anticoagulants: a 5-year quality improvement project.

[79] Field TS, Rochon P, Lee M (2009). Computerized Clinical Decision Support During Medication Ordering for Long-term Care Residents with Renal Insufficiency. J Am Med Inform Assoc.

[80] Petchey L, Gentry T (2019). More harm than good - why more isn’t always better with older people’s medicines.

[81] Hirst JA, Hill N, O’Callaghan CA (2020). Prevalence of chronic kidney disease in the community using data from OxRen: a UK population-based cohort study. Br J Gen Pract.

[82] Clews G (2023). NHS drug costs in England rose to more than £19bn in 2022/2023. Pharm J.

[83] Long CL, Raebel MA, Price DW (2004). Compliance with dosing guidelines in patients with chronic kidney disease. Ann Pharmacother.

[84] Dörks M, Allers K, Schmiemann G (2017). Inappropriate Medication in Non-Hospitalized Patients With Renal Insufficiency: A Systematic Review. J Am Geriatr Soc.

[85] MHRA (2019). Prescribing medicines in renal impairment: using the appropriate estimate of renal function to avoid the risk of adverse drug reactions.

[86] Foy R, Ovretveit J, Shekelle PG (2011). The role of theory in research to develop and evaluate the implementation of patient safety practices. BMJ Qual Saf.

[87] Tesfaye WH, Castelino RL, Wimmer BC (2017). Inappropriate prescribing in chronic kidney disease: A systematic review of prevalence, associated clinical outcomes and impact of interventions. Int J Clin Pract.

[88] Duncan P, Cabral C, McCahon D (2019). Efficiency versus thoroughness in medication review: a qualitative interview study in UK primary care. Br J Gen Pract.

[89] Stewart D, Whittlesea C, Dhital R (2020). Community pharmacist led medication reviews in the UK: A scoping review of the medicines use review and the new medicine service literatures. Res Social Adm Pharm.

[90] Taheri Moghadam S, Sadoughi F, Velayati F (2021). The effects of clinical decision support system for prescribing medication on patient outcomes and physician practice performance: a systematic review and meta-analysis. BMC Med Inform Decis Mak.

[91] Alagiakrishnan K, Ballermann M, Rolfson D (2019). Utilization of computerized clinical decision support for potentially inappropriate medications. Clin Interv Aging.

[92] van Marum RJ (2020). Underrepresentation of the elderly in clinical trials, time for action. Br J Clin Pharmacol.

