# Supplementary material for: Widespread inappropriate prescribing for older people with reduced kidney function: what are the harms and how do we tackle them? A scoping review for primary care
Source: BMJ Qual Saf. 2025 Oct 7;35(5):e018736. doi: 10.1136/bmjqs-2025-018736 (PMC13151456; doi:10.1136/bmjqs-2025-018736)
Supplement: online supplemental appendix 1 [file bmjqs-35-5-s001.pdf]

Appendices:

Appendix I – Mind map analysis of a case-note review undertaken by SW as part of her PHD thesis:

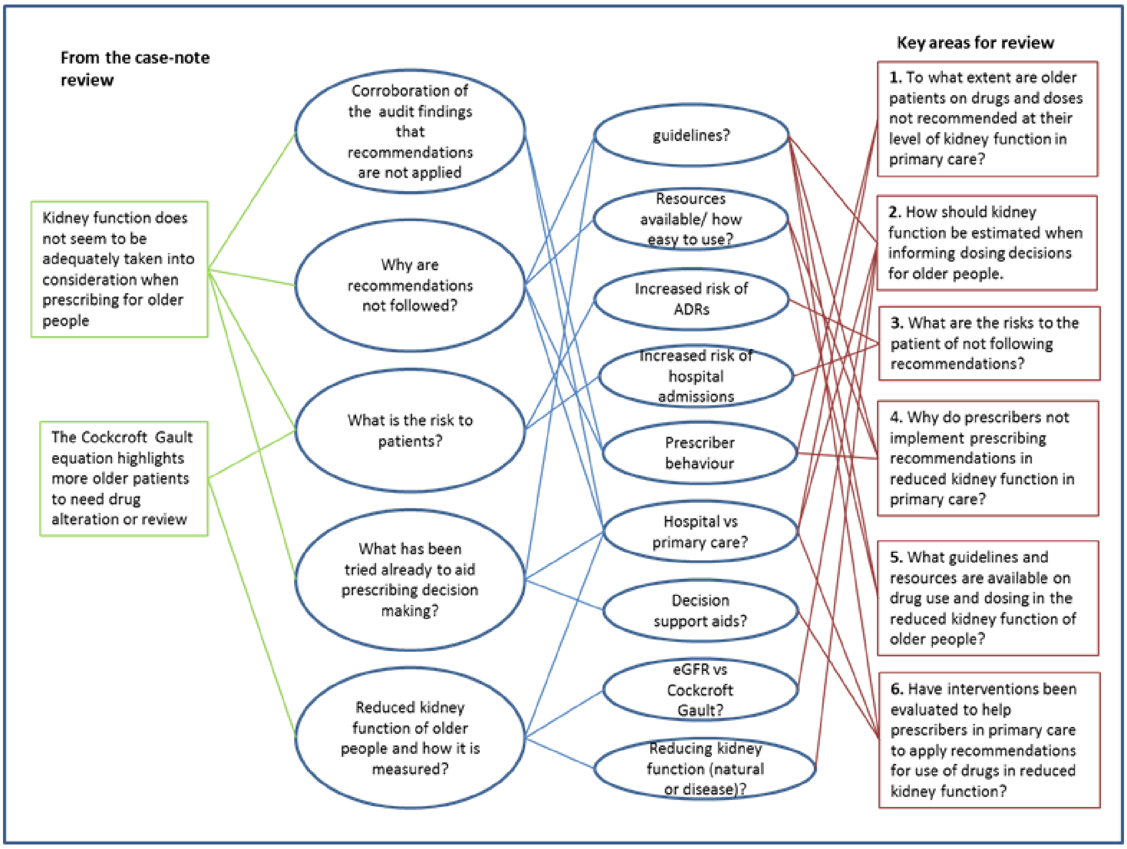

Abbreviations: ADRs: adverse drug reactions; eGFR: estimated glomerular filtration rate.

Reference: Wood SI. Are recommendations for prescribing applied for older people with reduced kidney function in primary care? A mixed methods study to explore and improve implementation. [Internet] [phd]. University of Leeds; 2016 [cited 2024 Nov 15]. Available from: https://etheses.whiterose.ac.uk/16504/

Figure reproduced with permission from Dr Su Wood.

Appendix II – Search strategy:

Review question 1 – To what extent are older people (≥65 years) on medications and doses not recommended at their level of kidney function in primary care?

- Eligibility criteria:
    - Participants:
        - Older people (≥65 years) or a population with 85% over age 65.
        - Taking drugs and doses not recommended at their level of kidney function.
        - Treated in primary care.
        - Assessed in terms of clinician compliance with prescribing recommendations in reduced kidney function.
    - Specific participant exclusion criteria:
        - Treated in secondary care.
        - Has not had their kidney function evaluated.
        - No assessment of clinician compliance with prescribing recommendations in reduced kidney function.
- Types of Sources:
    - Inclusion criteria:
        - Experimental and quasi-experimental study designs including randomized controlled trials, non-randomized controlled trials, before and after studies and interrupted time-series studies.
        - Analytical observational studies including prospective and retrospective cohort studies, case-control studies and analytical cross-sectional studies will be considered for inclusion.
        - Descriptive observational study designs including case series, individual case reports and descriptive cross-sectional studies will also be considered for inclusion.
    - Exclusion criteria:
        - Reviews will be reviewed for references but not included within the review.
        - Qualitative studies, along with text and opinion papers will be excluded.
- Search categories:
    - The search categories for this question where: Prescribing; Renal impairment; Elderly; Primary Care.

Review question 2 – What are the risks to the older person in primary care of not following the recommendations for prescribing in reduced kidney function?

- Eligibility criteria:
    - Participants:
        - Older people (≥65 years) or a population with 85% over age 65.

        - Taking drugs and doses not recommended at their level of kidney function.
        - Treated in primary care.
        - Intervention or outcome relating to assessment risks associated with not applying the prescribing recommendations in reduced kidney function.
    - Specific participant exclusion criteria:
        - Treated in secondary care.
        - Has not had their kidney function evaluated.
        - No assessment of risk for prescribing that does not follow recommendations in reduced kidney function.
- Types of Sources:
    - Inclusion criteria:
        - Experimental and quasi-experimental study designs including randomized controlled trials, non-randomized controlled trials, before and after studies and interrupted time-series studies will be considered.
        - Analytical observational studies including prospective and retrospective cohort studies, case-control studies and analytical cross-sectional studies will be considered for inclusion.
        - This review will also consider descriptive observational study designs including case series, individual case reports and descriptive cross-sectional studies for inclusion.
        - Only sources written in the English language will be included.
    - Exclusion criteria:
        - Reviews will be reviewed for references but not included within the review.
        - Qualitative studies, along with text and opinion papers will be excluded.
- Search categories:
    - The search categories for this question where: Renal impairment; Elderly; Primary Care; ADRs.

Review question 3 – Why do prescribers not apply prescribing recommendations in reduced kidney function in primary care?

- Eligibility criteria:
    - Participants:
        - Prescribing drugs to patients at a reduced level of kidney function.
        - Working in primary care.
    - Specific participant exclusion criteria:
        - Working in secondary care.
        - Not prescribing drugs to those with kidney impairment.
- Types of Sources:
    - Inclusion criteria:

        - Primary empirical qualitative research and reviews of primary empirical qualitative research will be considered.
        - Only sources written in the English language will be included.
    - Exclusion criteria:
        - Reviews will be reviewed for references but not included within the review.
        - Text and opinion papers will be excluded.
- Search categories:
    - The search categories for this question where: Renal impairment; Primary Care; Prescribers; Guidelines/recommendations; Prescribing; Prescriber Behaviour.

<u>Review question 4 – Have interventions been evaluated to help prescribers in primary care to apply recommendations for use of medications in older people with reduced kidney function?</u>

- Eligibility criteria:
    - Participants:
        - Intervention or outcome relating to evaluation of how to improve prescribing in reduced kidney function.
        - Implemented in primary care.
        - Older people (≥65 years) or a population with 85% over age 65.
    - Specific participant exclusion criteria:
        - Implemented in secondary care.
        - No intervention to improve prescribing in reduced kidney function.
- Types of Sources:
    - Inclusion criteria:
        - Experimental and quasi-experimental study designs including randomized controlled trials, non-randomized controlled trials, before and after studies and interrupted time-series studies will be included.
        - Analytical observational studies including prospective and retrospective cohort studies, case-control studies and analytical cross-sectional studies will be considered for inclusion.
        - Descriptive observational study designs including case series, individual case reports and descriptive cross-sectional studies will be considered for inclusion.
        - Only sources written in the English language will be included.
    - Exclusion criteria:
        - Qualitative studies, along with text and opinion papers, will be excluded.
- Search categories:
    - The search categories for this question where: Renal impairment; Primary Care; Decision support tools; Prescribing.

Search term variations:

Prescribers - P

- Subject heading:
  - Health Occupations explode - not PsychInfo
  - Health Personnel exp
- Keywords:
  - prescriber*
  - doctor*
  - GP*
  - general practitioner*
  - MD*
  - nurs*
  - physician*
  - phycisian*
  - (healthcare adj2 (professional* or worker* or staff))
  - (health adj3 (professional* or worker* or staff))
- CINAHL: prescriber*

Prescribing – Pg:

- Subject heading:
  - Drug Therapy explode
  - Drug Prescriptions exp
- Keywords:
  - prescrip*
  - prescrib*
  - 'drug use'
  - 'medication use'
  - drug dos*
  - medication dos*
  - medication order*
  - dos* adjustment

Guidelines/ recommendations – GR:

- Subject heading:
  - guideline or practice guideline not PsychInfo
  - guideline adherence not PsychInfo
- Keywords:
  - guideline*

  - guidance
  - guidance
  - recommendation*
  - ((guid* or recommendation*) adj4 (adher* or comply or complia* or complie* or follow* or observ*))
  - CINAHL: guideline or recommedation*

Primary Care – PC:

- Subject heading:
  - Primary Health Care CINAHL subject term
  - general practice or family practice not PsychInfo
- Keywords:
  - (primary adj (healthcare or health care or care))
  - general practice
  - GP*

Renal impairment – RI:

- Subject heading:
  - Renal Insufficiency or renal insufficiency, chronic not PsychInfo
- Keywords:
  - (renal adj (impairment, failure, dysfunction, or insufficiency))
  - (impaired adj (renal function or kidney function))
  - (reduced adj (renal function or kidney function or GFR or glomerular filtration rate or eGFR or creatinine clearance))
  - (kidney adj (failure or dysfunction))
  - (poor adj (renal function or kidney function))
  - (chronic adj (renal failure or kidney disease))
  - CKD
  - eGFR decline

Elderly – E:

- Subject heading:
  - Aged explode CINAHL subject term; aged or elderly
- Keywords:
  - elder*
  - over 65*
  - old* person*
  - old* patient*

ADRs – ADR:

- Subject heading:
    - Drug toxicity explode CINAHL subject term: adverse drug event or medication risk
    - Medication errors
- Keywords:
    - (drug adj2 (problem* or event* or error* or adverse))
    - (adverse adj3 (reaction* or event*))
    - ADR
    - (medication adj2 (error* or safety or adverse))
    - (renal risk adj (drug* or medication*))
    - Nephrotoxic
    - Hospital admission*
    - Hospitali*

Decision support tools – DS:

- Subject heading:
    - Decision Support Systems, Clinical
    - Decision Making, Computer-Assisted Therapy, Computer-Assisted
    - Decision Support Techniques
    - CINAHL subject term: decision support systems, clinical
- Keywords:
    - alert*
    - drug dos* service
    - dos* guid*
    - (compute* adj3 (support or tool* or service*))
    - (decision adj3 (support or tool* or service*))

Prescriber Behaviour – PB:

- Subject heading
    - N/A
- Keywords:
    - ((prescrib* or MD* or physician* or GP* or general practitioner* or nurse* or doctor*) adj (behavi* or performance or error))
    - ((prescrib* or MD* or physician* or GP* or general practitioner* or nurse* or doctor*) adj4 (knowledge or attitude* or belief* or barrier* or adhere* or implement* or compli*))
    - CINAHL: knowledge or attitude* or belief* or barrier* or adhere* or implement* or compli*

Appendix III – Data extraction tools:

To what extent are older people (≥65 years) on drugs and doses not recommended at their level of kidney function in primary care?

ID No.	Principal author	Hierarchy of evidence (based on OCEBM)	Year	Country	Study type	Setting	Population size	Sample size	Participant type	eGFR / CKD stage	Age	Associated condition	Medication identification	Type of guideline used	GFR estimation formulae used	Demographics	Specific medication / age / eGFR prevalence	Overall prevalence number	Drug or patient prevalence	Overall prevalence details	Search period	Quality grade
1																						
2																						

What are the risks to the older person in primary care of not following the recommendations for prescribing in reduced kidney function?

ID No.	Principal author	Hierarchy of evidence (based on OCEBM)	Year	Country	Study type	Setting	Population size	Sample size	Participant type	eGFR / CKD stage	Age	Associated condition	Medication identification	Type of guideline used	GFR estimation formulae used	Demographics	Overall harm number	Type of harm	summary outcome	Search period	Quality grade

Why do prescribers not apply prescribing recommendations in reduced kidney function in primary care?

ID No.	Principal author	Hierarchy of evidence	Year	Country	Study type	Setting	Focus	Participant type	Participant number (survey)	Participant number (interviews)	Themes	Outcome	Search period	Specific to reduced kidney function	Quality grade

Have interventions been evaluated to help prescribers in primary care to apply recommendations for use of drugs in reduced kidney function in older people?

ID No.	Principal author	Hierarchy of evidence (based on OCEBM)	Year	Country	Study type	Setting	Population size	Sample size	Participant type	eGFR / CKD stage	Age	Associated condition	main drug used	Primary intervention	Additional features / information	Type of guideline used	GFR estimation formulae used	Demographics	Effect	summary outcome	Search period	Quality score

Appendix IV - Preferred Reporting Items for Systematic Reviews and Meta-Analysis extension for Scoping Reviews (PRISMAScR) checklist:

SECTION	ITEM	PRISMA-ScR CHECKLIST ITEM	REPORTED ON PAGE #
**TITLE**			
Title	1	Identify the report as a scoping review.	1
**ABSTRACT**			
Structured summary	2	Provide a structured summary that includes (as applicable): background, objectives, eligibility criteria, sources of evidence, charting methods, results, and conclusions that relate to the review questions and objectives.	3
**INTRODUCTION**			
Rationale	3	Describe the rationale for the review in the context of what is already known. Explain why the review questions/objectives lend themselves to a scoping review approach.	4
Objectives	4	Provide an explicit statement of the questions and objectives being addressed with reference to their key elements (e.g., population or participants, concepts, and context) or other relevant key elements used to conceptualize the review questions and/or objectives.	4
**METHODS**			
Protocol and registration	5	Indicate whether a review protocol exists; state if and where it can be accessed (e.g., a Web address); and if available, provide registration information, including the registration number.	5
Eligibility criteria	6	Specify characteristics of the sources of evidence used as eligibility criteria (e.g., years considered, language, and publication status), and provide a rationale.	5; appendix I
Information sources*	7	Describe all information sources in the search (e.g., databases with dates of coverage and contact with authors to identify additional sources), as well as the date the most recent search was executed.	5; appendix I
Search	8	Present the full electronic search strategy for at least 1 database, including any limits used, such that it could be repeated.	5; appendix I

SECTION	ITEM	PRISMA-ScR CHECKLIST ITEM	REPORTED ON PAGE #
Selection of sources of evidence†	9	State the process for selecting sources of evidence (i.e., screening and eligibility) included in the scoping review.	5; appendix I
Data charting process‡	10	Describe the methods of charting data from the included sources of evidence (e.g., calibrated forms or forms that have been tested by the team before their use, and whether data charting was done independently or in duplicate) and any processes for obtaining and confirming data from investigators.	5; appendix I & III
Data items	11	List and define all variables for which data were sought and any assumptions and simplifications made.	5; appendix I & III
Critical appraisal of individual sources of evidence§	12	If done, provide a rationale for conducting a critical appraisal of included sources of evidence; describe the methods used and how this information was used in any data synthesis (if appropriate).	5
Synthesis of results	13	Describe the methods of handling and summarizing the data that were charted.	5; appendix I & III
**RESULTS**			
Selection of sources of evidence	14	Give numbers of sources of evidence screened, assessed for eligibility, and included in the review, with reasons for exclusions at each stage, ideally using a flow diagram.	6; appendix II
Characteristics of sources of evidence	15	For each source of evidence, present characteristics for which data were charted and provide the citations.	7,9,10,11
Critical appraisal within sources of evidence	16	If done, present data on critical appraisal of included sources of evidence (see item 12).	7,9,10,11
Results of individual sources of evidence	17	For each included source of evidence, present the relevant data that were charted that relate to the review questions and objectives.	13-20
Synthesis of results	18	Summarize and/or present the charting results as they relate to the review questions and objectives.	7,9,10,11

SECTION	ITEM	PRISMA-ScR CHECKLIST ITEM	REPORTED ON PAGE #
**DISCUSSION**			
Summary of evidence	19	Summarize the main results (including an overview of concepts, themes, and types of evidence available), link to the review questions and objectives, and consider the relevance to key groups.	21
Limitations	20	Discuss the limitations of the scoping review process.	22
Conclusions	21	Provide a general interpretation of the results with respect to the review questions and objectives, as well as potential implications and/or next steps.	22
**FUNDING**			
Funding	22	Describe sources of funding for the included sources of evidence, as well as sources of funding for the scoping review. Describe the role of the funders of the scoping review.	1

JBI = Joanna Briggs Institute; PRISMA-ScR = Preferred Reporting Items for Systematic reviews and Meta-Analyses extension for Scoping Reviews.

* Where *sources of evidence* (see second footnote) are compiled from, such as bibliographic databases, social media platforms, and Web sites.

† A more inclusive/heterogeneous term used to account for the different types of evidence or data sources (e.g., quantitative and/or qualitative research, expert opinion, and policy documents) that may be eligible in a scoping review as opposed to only studies. This is not to be confused with *information sources* (see first footnote).

‡ The frameworks by Arksey and O'Malley (6) and Levac and colleagues (7) and the JBI guidance (4, 5) refer to the process of data extraction in a scoping review as data charting.

§ The process of systematically examining research evidence to assess its validity, results, and relevance before using it to inform a decision. This term is used for items 12 and 19 instead of "risk of bias" (which is more applicable to systematic reviews of interventions) to include and acknowledge the various sources of evidence that may be used in a scoping review (e.g., quantitative and/or qualitative research, expert opinion, and policy document).

From: Tricco AC, Lillie E, Zarin W, O'Brien KK, Colquhoun H, Levac D, et al. PRISMA Extension for Scoping Reviews (PRISMAScR): Checklist and Explanation. Ann Intern Med. 2018;169:467–473. doi: 10.7326/M18-0850.

Appendix V – Review question-specific PRISMA flow charts:

Review question 1 – To what extent are older people (≥65 years) on medicines and doses not recommended at their level of kidney function in primary care?

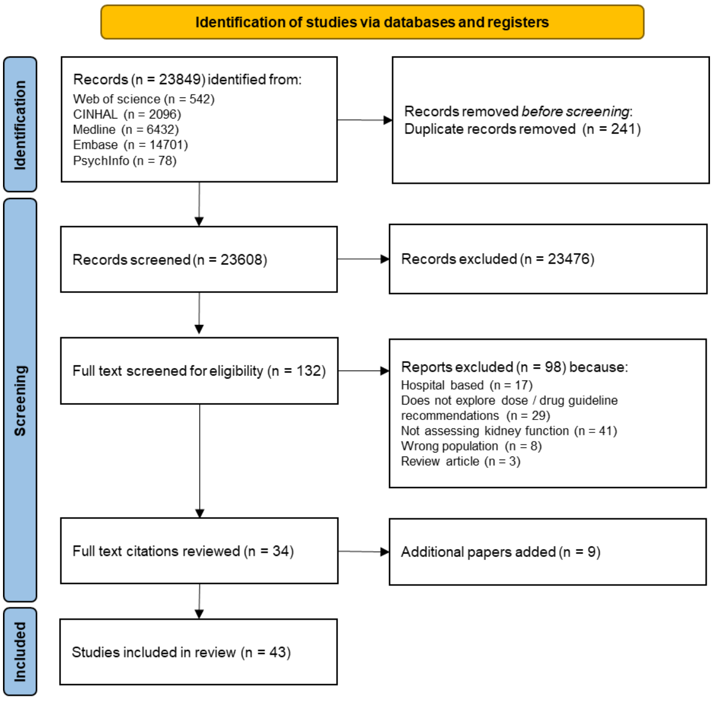

Diagram adapted from: Page MJ, et al. BMJ 2021;372:n71. doi: 10.1136/bmj.n71.
This work is licensed under CC BY 4.0. To view a copy of this license, visit https://creativecommons.org/licenses/by/4.0/

Review question 2 – What are the risks to the older person in primary care of not following the recommendations for prescribing in reduced kidney function?

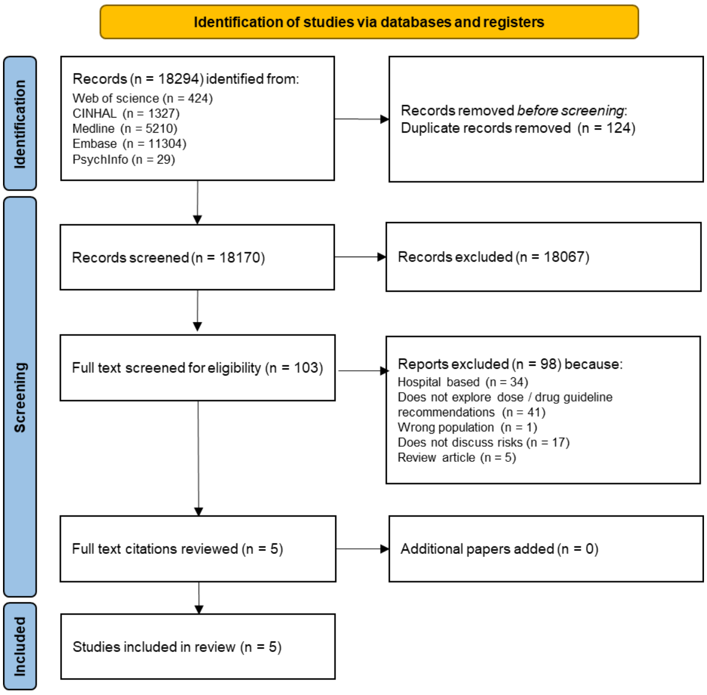

Diagram adapted from: Page MJ, et al. BMJ 2021;372:n71. doi: 10.1136/bmj.n71.
This work is licensed under CC BY 4.0. To view a copy of this license, visit https://creativecommons.org/licenses/by/4.0/

Review question 3 – Why do prescribers not apply prescribing recommendations in reduced kidney function in primary care?

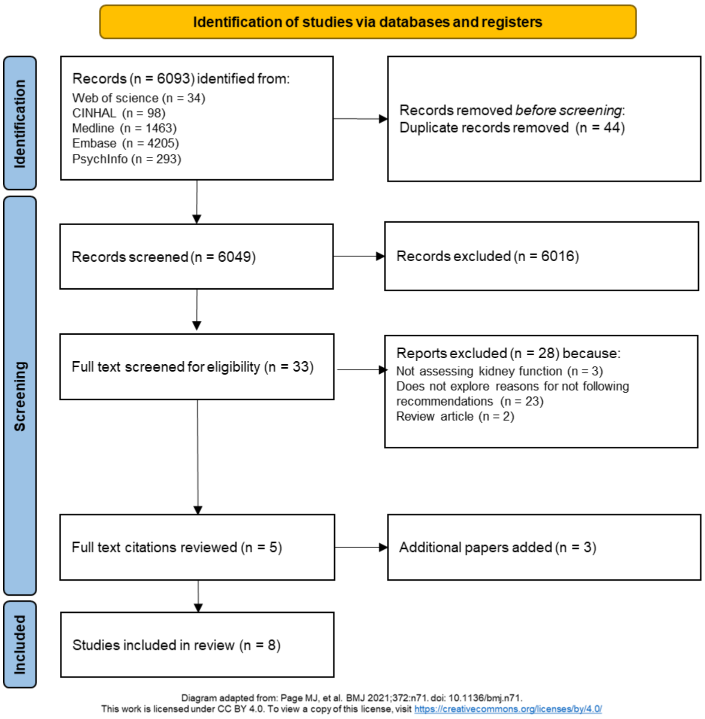

Review question 4 – Have interventions been evaluated to help prescribers in primary care to apply recommendations for use of medications in older people with reduced kidney function?

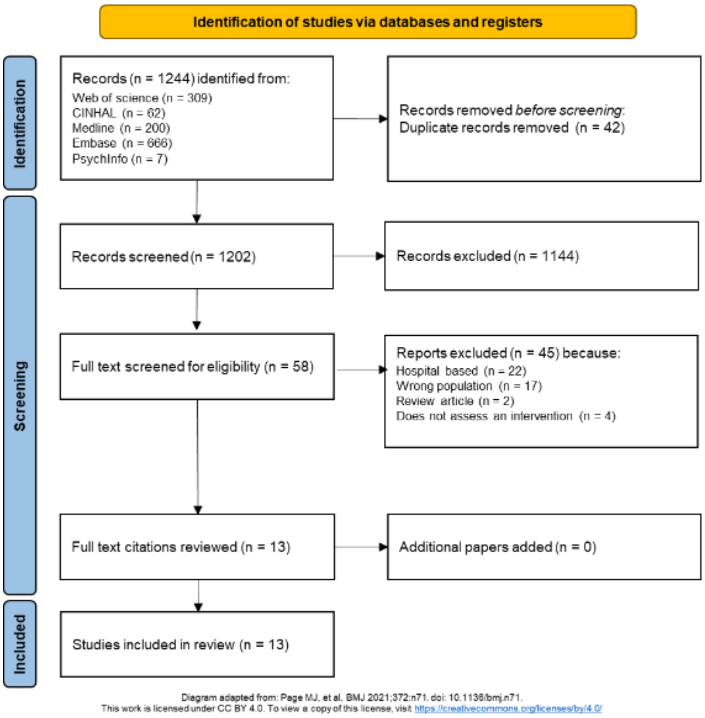

Appendix VI – Included studies, study characteristics, and results (listed by research question and sorted by publication date)

Question 1 – What is the scale of the problem in primary care?										
**Principal author and year**	**Country and setting**	**Sample size**	**Study type**	**Participant characteristics**	**Medication identification tool**	**Prescribing guideline source**	**GFR estimation formulae**	**Overall prevalence**	**Medication or patient prevalence**	**Quality grade**
**Papaioannou et al., 2000** [42]	Canada – care home	456	Cross-sectional	CKD; Age >65	List (Renally excluded medications from consultation with specialists).	Drug Prescribing in Renal Failure: Dosing Guidelines for Adults; Geriatric Dosing Handbook; Compendium of Pharmaceuticals and Specialties Ottawa; AHFS Drug Information	Cockcroft-Gault formula	43.2%	Patient	High
**Rothberg et al., 2008** [43]	USA – primary care	814	Cross-sectional	Age >65	Medication review	Not stated	eGFR (MDRD)	10.2%	Patient	High
**Breton et al., 2011** [25]	France – primary care	8701	Cross-sectional and cohort	Age >65	Medication review	Principal French drug dictionaries (Vidal and Dorosz), as well as from Information Conseil Adaptation Renale (ICAR) website	eGFR (MDRD)	13.3%	Patient	High
**Hanlon et al., 2011** [44]	USA – VA care home	1304	Cross-sectional	Age >65	List - 21 drugs developed by consensus using a Delphi survey and guidelines	Developed by consensus using a Delphi survey; VA CKD guidelines	Cockcroft-Gault formula; eGFR (MDRD)	6.0%	Patient	High
**Wood et al., 2011** [45]	UK – primary care	594	Cross-sectional	Age >65	Medication review	SmPC; BNF	Cockcroft-Gault formula	25.0%	Patient	Acceptable
**Erler et al., 2012** [28]	Germany – primary care	206	RCT	GFR <50; or Age >70	Medication review	The software programme "DOSING"	Not stated	34.5%	Patient	Acceptable
**Schmidt-Mende et al., 2012** [46]	Sweden – primary care	3345	Cross-sectional	CKD; Age >65; hypertension	Medication review	Not stated	Cockcroft-Gault formula	n/a	n/a	Acceptable
**Durand et al., 2013** [47]	USA – primary care	52	Cross-sectional	GFR <51	Renally excreted antibiotics	LexiComp; Drug Prescribing in Renal Failure	Cockcroft-Gault formula	27.0%	Medication	Low

**Joosten et al., 2013** [29]	Netherlands – primary care	1369	Single arm intervention study	GFR <40	Medication review	Dutch - ‘the National Formulary on drug prescribing in renal impairment’; ‘National Shared Care Guidelines on Chronic Kidney Disease (CKD)’	eGFR (MDRD)	15.0%	Patient	Acceptable
**Via-Sosa et al., 2013** [30]	Spain – primary care	263	Cross-sectional	Age >65; polypharmacy	Medication review	CIMA (Medicines Online Information Centre); the Spanish official drugs Information; Martindale and American Hospital Formulary Drug System Information	Cockcroft-Gault method and eGFR (MDRD) - unclear when each used	17.5%	Patient	Acceptable
**Barnes et al., 2014** [31]	USA – care home	146	Before and after study	CKD	Medication review	Micromedex online database	Cockcroft-Gault formula	7.2%	Medication	Low
**Farag et al., 2014** [32]	Canada – primary care	1464	Case series	CKD 4-5; Age >65	List of antibiotics	Canadian Compendium of Pharmaceuticals and Specialties	eGFR (MDRD)	27.0%	Medication	Acceptable
**Gheewala et al., 2014** [33]	Australia – care home	323	Cross-sectional	CKD	Medication review	Australian Medicines Handbook	eGFR (MDRD)	8.7%	Patient	Low
**Steinman et al., 2014** [48]	USA – VA primary care	462,405	Cross-sectional	Age >65	Medication review	Lexicomp	eGFR (CKD-EPI)	6.1%	n/a	Acceptable
**Van Pottelbergh et al., 2014** [49]	Belgium – primary care	539	Cross-sectional	Age >80	Medication review	SmPC; Dutch guideline and a Belgian drug database	eGFR (MDRD)	8.2%	Patient	Acceptable
**Khanal et al., 2015** [50]	Australia – Primary care and care home	4035	Cross-sectional	Age >65	Medication review - 31 selected drugs	Australian Medicines Handbook	Cockcroft-Gault formula	28.1%	Patient	Acceptable

					from the Department of Veterans' Affairs					
**Chang et al., 2015** [51]	USA – VA primary care	83,850	Cross-sectional	GFR 15-49; Age >65	Medication review	LexiComp; a consensus report of medications that require dose adjustment in older adults.	Cockcroft-Gault formula	15.0%	Patient	High
**Pourrat et al., 2015** [34]	France – primary care	177	Cross-sectional	Age >65; Hypertension or T2DM	2 or more drugs for hypertension or T2DM	Expert group	eGFR (MDRD)	24.9%	Patient	High
**Becquemont et al., 2016** [26]	France – primary care	588	Cross-sectional	Age >65; T2DM	Metformin	SmPC; ISS	eGFR (CKD-EPI)	21.9%	Patient	High
**Hoffmann et al., 2016** [52]	Germany – Care home	685	Cross-sectional	Care home resident	Medication review	SmPC	Cockcroft-Gault formula	19.7%	Both	Acceptable
**Koster et al., 2016** [53]	Netherlands – primary care	156	Cross-sectional	Age >65	Medication review	Dutch College of General Practitioners guidelines	eGFR (MDRD)	0.6%	Patient	Low
**Parbtani et al., 2016** [54]	Canada – primary care	20	Cross-sectional	Age >75	DOAC	Not stated	eGFR (MDRD) and Cockcroft-Gault formula	40.0%	Patient	Low
**Pascart et al., 2016** [55]	France – primary care	349	Cross-sectional	Gout	Colchicine	2006 EULAR recommendations	Cockcroft-Gault formula	18.6%	Medication	High
**Tebboth et al., 2016** [56]	UK – primary care	3425	Cross-sectional	Age >40; Gout	DPP-4	SmPC	eGFR (MDRD)	23.0%	Patient	Acceptable
**Trifiro et al., 2016** [57]	Italy – primary care	725	Cohort	CKD; T2DM	Medication review	SmPC; the kidney disease outcomes quality initiative (K-DOQI) guidelines	Cockcroft-Gault formula	32.5%	Patient	High

**Khanal et al., 2017** [58]	Australia – primary care	2628	Cross-sectional	n/a	Medication review - 31 selected drugs from the Department of Veterans' Affairs, Australia	Australian Medicines Handbook	Cockcroft-Gault formula, eGFR (MDRD, MDRD weight, CKD-EPI, CKD-EPI weight)	12.9%	Medication	High
**Guirguis-Blake et al., 2018** [59]	USA – primary care	172	Cross-sectional	CKD 3-4	NSAIDs; list from the family medicine literature of contraindicated or dose adjustment	A published list in the family medicine literature (NSAIDs, acarbose, chlorpropamide, glyburide and nitrofurantoin)	Not stated	31.7%	Patient	Low
**Schmidt-Mende et al., 2018** [60]	Sweden – primary care	32,533	Cross-sectional	CKD; Age >65	Medication review	Swedish "Janusmed Drugs and Renal function."	eGFR (CKD-EPI)	42.5%	Patient	High
**Wood et al., 2018** [12]	UK – primary care	549 533	Cross-sectional	Age >65	Medication review	NICE; BNF; SmPC	Cockcroft-Gault formula	n/a	n/a	High
**Manski-Nankervis et al., 2019** [61]	Australia – primary care	3505	Cross-sectional	CKD; T2DM	Non-insulin diabetes medication	Australian Diabetes Society (ADS) guidelines	Not stated	n/a	n/a	Acceptable
**Spanopoulos et al., 2019** [62]	UK – primary care	2580	Cross-sectional	T2DM	DPP-4	SmPC	Cockcroft-Gault formula or eGFR (CKD-EPI)	45.0%	Patient	Acceptable
**Zhu et al., 2019** [63]	Canada – primary care	3937	Case-control	GFR <30; Age >65	Antibiotic	UpToDate'; Compendium of Pharmaceuticals and Specialties (CPS)	eGFR (CKD-EPI)	18.6%	Medication	Low
**Bezabhe et al., 2020** [64]	Australia – primary care	44,259	Cross-sectional	CKD 3-4	List of drugs from Smits et al. - 16 indicators	Smits et al. - 16 prescribing quality indicators (PQIs)	eGFR (CKD-EPI)	n/a	n/a	High
**Cardoso et al., 2020** [65]	Portugal – primary care	772	Cross-sectional	AF	DOAC	SmPC	Cockcroft-Gault formula	31.2%	Patient	High
**Castelino et al., 2020** [66]	Australia – primary care	48,731	Cross-sectional	CKD	Medication review	Australian Medicines Handbook	Cockcroft-Gault formula; eGFR (CKD-EPI)	35.0%	Patient	High

**Ferrat et al., 2021** [67]	France – primary care	1111	Cross-sectional	AF	DOAC	Predefined by an expert group on the basis of the SmPC	Cockcroft-Gault formula	n/a	n/a	Acceptable
**MacRae et al., 2021** [68]	UK – primary care	23292	Cohort	CKD	Medication review	BNF	eGFR (MDRD)	22.2%	Patient	High
**Silva-Almodóvar et al., 2021** [69]	USA – primary care	3624	Cross-sectional	CKD	Medication review	Lexicomp	eGFR (CKD-EPI)	33.0%	Patient	Acceptable
**Troncoso-Mariño et al., 2021** [27]	Spain – primary care	723,016	Cross-sectional and cohort	Age >65	Medication review	Catalan Health Department's consensus for patients with CKD	eGFR (MDRD)	11.1%	Patient	Acceptable
**Bezabhe et al., 2022** [70]	Australia – primary care	11,251	Cross-sectional	AF	DOAC	Current Australian AF guideline	Cockcroft-Gault formula	n/a	n/a	High
**Ramos et al., 2022** [35]	Spain – Community pharmacy	179	Single arm intervention study	Age >60	Medication review	Community pharmacist knowledge	eGFR (CKD-EPI)	39.1%	Patient	Low
**Ruiz-Boy et al., 2022** [71]	Spain – primary care	273	Cross-sectional	CKD	Medication review	SmPC; Micromedex; UpToDate	eGFR (CKD-EPI)	49.1%	Patient	High
**Naghnaghia et al., 2023** [72]	Palestine – primary care	421	Cross-sectional	Age >60	List from the beers criteria	The Beers criteria	Not stated	36.8%	Patient	Acceptable
**Question 2 – What is the risk of harm to older patients?**										
**Principal author and year**	**Country and setting**	**Sample size**	**Study type**	**Participant characteristics**	**Medication identification tool**	**Prescribing guideline source**	**GFR estimation formulae**	**Overall harm**	**Type of harm**	**Quality grade**
**Helldén et al., 2009** [39]	Sweden – Emergency department	154	Cohort	Age >65	Not stated	Janusinfo (the Swedish version of the Physician's Desk Reference)	Cockcroft-Gault formula; eGFR (MDRD)	OR 1,493	Hospital admission	Acceptable

**Breton et al., 2011** [25]	France – Primary care	8701	Cross-sectional and cohort	Age >65	Medication review	Principal French drug dictionaries (Vidal and Dorosz), as well as from Information Conseil Adaptation Renale (ICAR) website	eGFR (MDRD)	Non-significant result	Mortality (HR)	Acceptable
**Becquemont et al., 2016** [26]	France – Primary care	588	Cross-sectional	Age >; T2DM	Metformin	SmPC; ISS	eGFR (CKD-EPI)	Non-significant result	Mortality (HR)	High
**Alarkawi et al., 2020** [38]	UK and Spain – Primary care	320578	Cohort	GFR <45; Age >40; Osteoporosis	Bisphosphonates	SmPC	Not stated	(-)30 to 0%	Mortality (HR)	Acceptable
**Troncoso-Mariño et al., 2021** [27]	Spain – Primary care	723,016	Cross-sectional and cohort	Age >65	Medication review	Catalan Health Department’s consensus recommendation for patients with CKD	eGFR (MDRD)	6-8%	Mortality (HR)	High

Question 3 – Why is it difficult to follow prescribing guidelines in reduced kidney function?

**Principal author**	**Country and participant type**	**Focus**	**Study type**	**Themes**	**Focused on kidney function?**	**Quality grade**
**Bradley et al., 1992** [73]	UK – GP	Prescribing decisions	Semi-structured interviews	Balancing other considerations; Lack of awareness; Concern about toxicity	No	Acceptable
**Jonville-Béra et al., 2008** [74]	France – GP	Prescribing behaviour in hidden renal dysfunction	Qualitative survey	Concerns about accessing / trusting / using guidelines in primary care; Lack of awareness	Yes	Acceptable
**Wood et al., 2016** [18]	UK – GP	To systematically identify the potential barriers and enablers to prescribing appropriately in reduced kidney function using the Theoretical Domains Framework (TDF).	Semi-structured interviews	Lack of awareness; Confusion regarding different kidney function formulas; Balancing other considerations; Warning overload; Concerns about accessing / trusting / using guidelines in primary care; Concern about toxicity	Yes	High

**Schmidt-Mende, 2019** [36]	Sweden – GP; Nurse	To understand GPs' and nurses' views on potentially inappropriate prescribing and medication reviews	Semi-structured interviews	Complexity in prescribing in the elderly; Concerns about accessing / trusting / using guidelines in primary care; Threats to GP autonomy	Yes	Low
**Teh et al., 2019** [75]	Malaysia – Pharmacist	To explore pharmacists' self-perceived knowledge, attitude, and practice towards dosage adjustment among CKD patients	Qualitative survey	Concerns about accessing / trusting / using guidelines in primary care; Lack of awareness	Yes	Acceptable
**Campbell et al., 2020** [76]	Canada – GP	To explore the perspectives of general practitioners (GPs) regarding interventions to increase initiation of cholesterol lowering medication (or statins)	Semi-structured interviews	Confusion regarding different kidney function formulas; Confusion about prescribing in the elderly; Lack of awareness	No	Acceptable
**Allouchery et al., 2021** [77]	France – GP	Assess the knowledge of GPs about metformin-related Lactic Acidosis (LA), its prevention and to evaluate the information given to their patients.	Qualitative survey	Lack of awareness; Improvement due to external support	No	Low
**Flory et al., 2022** [78]	USA – Endocrinologists; General internists; Nurse practitioner; Primary care provider	To better understand how providers choose antidiabetic drugs in T2D with CKD	Semi-structured interviews and qualitative survey	Concerns about accessing / trusting / using guidelines in primary care; Concerns about toxicity; Lack of awareness	Yes	Acceptable

Question 4 – What has been shown to help improve prescribing in reduced kidney function?

Principal author	Country and setting	Sample size	Study type	Patient characteristics	Primary intervention	Additional features / information	Prescribing guideline source	GFR estimation formulae	Intervention effect	Quality score
**Field et al., 2009** [40]	Canada – Care home	833	RCT	Taking a medication from a list of risky medications	CDSS	n/a	Geriatric and psychotropic drug dosing handbooks and the Micromedex® online knowledge base	Cockcroft-Gault formula	Positive trend	Acceptable

**Erler et al., 2012** [28]	Germany – Primary care	404	RCT	GFR <50 or Age >70; Any medication	CDSS	Multi-faceted intervention - 1) education; 2) checklist; 3) leaflet	The software is continuously updated by the Department of Clinical Pharmacology and Pharmacoepidemiolog y, University Hospital Heidelberg, Germany.	Not stated	Positive	Acceptable
**Geerts et al., 2012** [79]	Netherlands – Primary care	650	Single arm study	Age >70; Diabetes or CVD; Taking a medication from a list of diabetic and CVD medications	Pharmacist medication review & CDSS	n/a	Dutch guidelines for drug administration in reduced renal function	eGFR (MDRD)	Positive trend	Low
**Joosten et al., 2013** [29]	Netherlands – Primary care	1,369	Single arm study	Any medication	CDSS	Weekly automatically generated a report for any ambulatory patients with an eGFR ≤40 ml/min/1.73 m2 for the pharmacists.	Dutch Pharmacists' guidelines including 'the National Formulary on drug prescribing in renal impairment' and the 'National Shared Care Guidelines on Chronic Kidney Disease (CKD)'	eGFR (MDRD)	Positive trend	Acceptable
**Via-Sosa et al., 2013** [30]	Spain – Community pharmacy	354	Historical control randomised study	Age >65; Polypharmacy; Any medication	Pharmacist medication review	A multicentre interventional study involving recommendations to physicians by pharmacists who have been given 2 hours training.	CIMA (Medicines Online Information Centre); the Spanish official drugs Information; Martindale and American Hospital Formulary Drug System Information (AHFS)	Cockcroft-Gault formula and eGFR (MDRD) - unclear when each used	Positive	High

**Barnes et al., 2014** [31]	USA – Care home	146	Before and after study	CKD; Any medication	Pharmacist medication review	n/a	Micromedex online database	Cockcroft-Gault formula	Positive trend	Low
**Farag et al., 2014** [32]	Canada – Primary care	1,464	Time series analysis	CKD 4/5; Age >65; Taking a medication from a list of antibiotics	eGFR reporting in ambulatory laboratories	n/a	Canadian Compendium of Pharmaceuticals and Specialties	eGFR (MDRD)	Neutral	Acceptable
**Gheewala et al., 2014** [33]	Australia – Care home	323	Single arm study	Any medication	Pharmacist medication review	n/a	Australian Medicines Handbook	Cockcroft-Gault formula	Positive trend	Low
**Pourrat et al., 2015** [34]	Community pharmacy	180	Single arm study	Age >65; Diabetes or hypertension; Any medication	Pharmacist medication review	Education	A team of experts - nephrologists and hospital clinical pharmacists.	eGFR (MDRD)	Positive trend	Acceptable
**Keohane et al., 2017** [41]	France – Primary care	158	Before and after study	Taking an NSAID	CDSS	A medication alert on each patients' individual medical record that displayed when a drug from the ATC code MO1A (NSAID class) relative to the patients' most recent eGFR.	Not stated	eGFR (MDRD)	Positive trend	Low
**Schmidt-Mende, 2019** [36]	Sweden – Primary care	69	RCT (cluster)	Age >65; Taking a medication from the from STOPP/START Criteria V2	Education		STOPP/START Criteria V2	eGFR (CKD-EPI)	Neutral	Acceptable

						A multifaceted educational intervention				
**Ramos et al., 2022** [35]	Spain – Community pharmacy	179	Single arm study	Age >60; Any medication	Pharmacist medication review	Glomerular filtration rate (eGFR) is calculated using CKD-EPI using the StatSensor Express POC meter	Community pharmacist knowledge	eGFR (CKD-EPI)	Positive trend	Low
**Jones et al., 2023** [80]	UK – Primary care	577	Time series analysis	AF; Taking a DOAC	Pharmacist medication review & CDSS	Initially, education sessions and information leaflets were provided to prescribers. Subsequently, a popup alert system was integrated Finally, the involvement of community pharmacists to evaluate patients	Not stated	Cockcroft-Gault formula	Positive trend	Low

Abbreviations: GFR (Glomerular Filtration Rate); CKD (Chronic Kidney Disease); AHFS (American Hospital Formulary Service); USA (United States of America); eGFR (estimated Glomerular Filtration Rate); MDRD (Modification of Diet in Renal Disease); VA (Veterans Affairs); UK (United Kingdom); SmPC (Summary of Product Characteristics); BNF (British National Formulary); RCT (Randomised Controlled Trial); CKD-EPI (Chronic Kidney Disease Epidemiology Collaboration); T2DM (Type 2 Diabetes Mellitus); ISS (Integrated Summary of Safety); DOAC (Direct Oral Anti-Coagulant); EULAR (European Alliance of Associations for Rheumatology); DPP-4 (dipeptidyl Peptidase-4); AF (Atrial Fibrillation); OR (Odds Ratio); HR (Hazard Ratio); GP (General Practice); CDSS (Clinical Decision Support System); NSAID (Non-Steroidal Anti-Inflammatory Drug); ATC (Anatomical Therapeutic Chemical); POC (Point Of Care).

Where the term ‘drug’ has been used in this table, it refers to the reference from the original wording in the related study and is the equivalent of the term ‘medication’ that is used preferentially throughout the main body of this publication.
